# Entropic Phenomena in Spin-Ice Materials and Magnetic Monopole Excitations

**DOI:** 10.3390/e28091039

**Published:** 2026-09-21

**Authors:** Andres Chappa, Ludi Miao

**Affiliations:** Department of Physics, New Mexico State University, Las Cruces, NM 88001, USA

**Keywords:** entropy, statistical physics, magnetic frustration, spin ice, magnetic monopoles

## Abstract

Spin ice provides a canonical setting in which geometric frustration, local constraints, and extensive degeneracy generate nontrivial entropy and emergent quasiparticles. In this review, we examine entropic phenomena in spin-ice materials and related pyrochlore magnets, taking entropy as a unifying theme that connects established spin-ice thermodynamics with recent developments in entropy-driven phases, monopole confinement, and interface-engineered monopole matter. We first review the connection between proton disorder in water ice and the ice-rule manifold of pyrochlore spin ice, including the Pauling residual entropy and its calorimetric observation, and then contrast this classical limit with quantum spin ice and lithographically constructed artificial spin ice. We next discuss pyrochlore iridates, where competition between the intrinsic spin-ice interaction and the Ir-induced local exchange field produces two-in–two-out (2I2O), fragmented, and all-in–all-out (AIAO) states with distinct residual entropies, as well as finite-temperature entropy-enhanced phases near competing ground-state boundaries. We next review emergent magnetic monopoles in spin ice, including monopole creation, Dirac strings, magnetic Coulomb interactions, and key experimental signatures of magnetic charge, current, and noise. Building on this framework, we discuss theoretically proposed two-dimensional magnetic monopole gases at spin-ice/pyrochlore iridate interfaces, where boundary conditions stabilize a net magnetic charge and entropy controls the spatial confinement of the monopole gas. Finally, we consider proposed monopole-based devices, including a magnetic field-controlled monopole transistor and position-based monopole traps. By linking classical residual entropy to entropy-driven phase selection, quasiparticle confinement, and device concepts, this review highlights how entropy can evolve from a thermodynamic signature of frustration into a potential design parameter for emergent magnetic matter.

## 1. Introduction

Frustrated magnetic systems arise when competing interactions, lattice geometry, or local constraints prevent all microscopic interactions from being simultaneously minimized [1,2,3]. Instead of selecting a unique conventionally ordered ground state, such systems can retain a large manifold of low-energy configurations accompanied by strong correlations, fluctuations, and emergent collective behavior [4,5]. Frustration therefore changes the usual balance between energy and entropy: configurational degeneracy can remain relevant deep into the low-temperature regime and can become a decisive contribution to the thermodynamics. Frustration can also be engineered deliberately in artificial nanomagnet arrays and programmable quantum simulators [6,7,8,9,10].

Frustration can originate from several mechanisms. Geometric frustration occurs when lattice connectivity prevents all local interactions from being satisfied simultaneously, as in triangular, kagome, and pyrochlore systems [1,6,11,12]. Competing exchange interactions and correlated-electron effects provide additional routes to frustration [1,3,13], while spin-orbit coupling [14], anisotropic exchange [15], orbital degrees of freedom [16], and long-range dipolar interactions [17] further enrich the phase space. Pyrochlore magnets [15,18] are particularly important because their corner-sharing tetrahedral geometry supports a broad range of frustrated states, including spin ice [19], Coulomb phases [20], magnetic fragmentation [21], and emergent magnetic monopoles [22].

A central thermodynamic consequence of frustration is residual entropy [18,19]. In a conventional ordered magnet, the number of thermally accessible configurations collapses as the temperature is lowered. In a frustrated magnet, local constraints may instead leave a macroscopically large number of configurations nearly or exactly degenerate, producing finite configurational entropy in the low-temperature regime. This entropy is not random disorder; it reflects correlated many-body constraints and can reshape the free energy landscape. Spin ice provides the canonical example: the local ice rule generates an extensively degenerate manifold analogous to proton disorder in water ice and yields the magnetic analogue of the Pauling residual entropy [19,23,24].

Pyrochlore iridates provide a complementary setting in which this entropy can be partially lifted, redistributed, or enhanced by competing magnetic interactions [21,25]. The ordered Ir sublattice generates a local exchange field on the rare-earth moments, producing a progression from the spin-ice manifold to a fragmented phase and ultimately to AIAO order. Near the boundaries between these states, competing configurations can generate finite-temperature phases with entropy exceeding that of either neighboring ground state [25]. Experimental work on ${\mathrm{Er}}_{2}$${\mathrm{Ir}}_{2}$$O_{7}$ has also reported a magnetic anomaly near 140 K together with a measurable magnetocaloric entropy change, providing a complementary thermodynamic perspective on pyrochlore iridates [26]. These systems therefore provide a direct route for examining how entropy participates in phase selection rather than merely characterizing the resulting state.

The same constrained manifold also supports a second major consequence of frustration: emergent magnetic monopoles [22,27,28,29]. Local violations of the ice rule fractionalize into effective magnetic charges that can separate through successive spin flips, interact through a magnetic Coulomb potential, and remain connected by Dirac strings [22,30]. Experimental signatures of these excitations have been reported through neutron scattering [20,27], magnetic charge and current measurements [28,31], susceptibility [32], and magnetic-noise spectroscopy [29]. Their existence demonstrates how local constraints in a frustrated magnet can generate quasiparticles with properties that have no counterpart among the microscopic spins.

Taken together, these developments motivate the central narrative of this review: a progression from frustration and residual entropy to emergent monopoles and, ultimately, entropy-engineered monopole matter. We first review classical spin ice and the thermodynamic origin and experimental observation of residual entropy and then introduce quantum and artificial spin ice as complementary extensions of the ice-rule framework. We then discuss pyrochlore iridates and entropy-enhanced phases produced by competing magnetic states. Next, we review emergent monopoles, Dirac strings, and their principal experimental signatures. Finally, we examine two-dimensional magnetic monopole gases in oxide heterostructures, where interface boundary conditions create a net magnetic charge and entropy determines the spatial confinement of the quasiparticles, followed by proposed device concepts and future directions for entropy engineering in frustrated materials.

## 2. Spin Ice and Residual Entropy

Spin ice is a canonical example of geometrically frustrated magnetism and of residual entropy in a magnetic system [19,24]. Its connection to proton disorder in water ice [19,23] provides a direct statistical-mechanical picture of how a local constraint can generate an extensively degenerate manifold. The resulting ice-rule states retain finite configurational entropy [24] and support emergent phenomena including Coulomb-phase correlations [20] and fractionalized magnetic monopole excitations [22]. We therefore begin with the origin and thermodynamic observation of the classical spin-ice residual entropy before considering how the ice-rule framework is extended by quantum fluctuations and by artificial nanomagnetic architectures.

### 2.1. Water Ice, Proton Disorder, and Pauling Entropy

**Figure 1:**
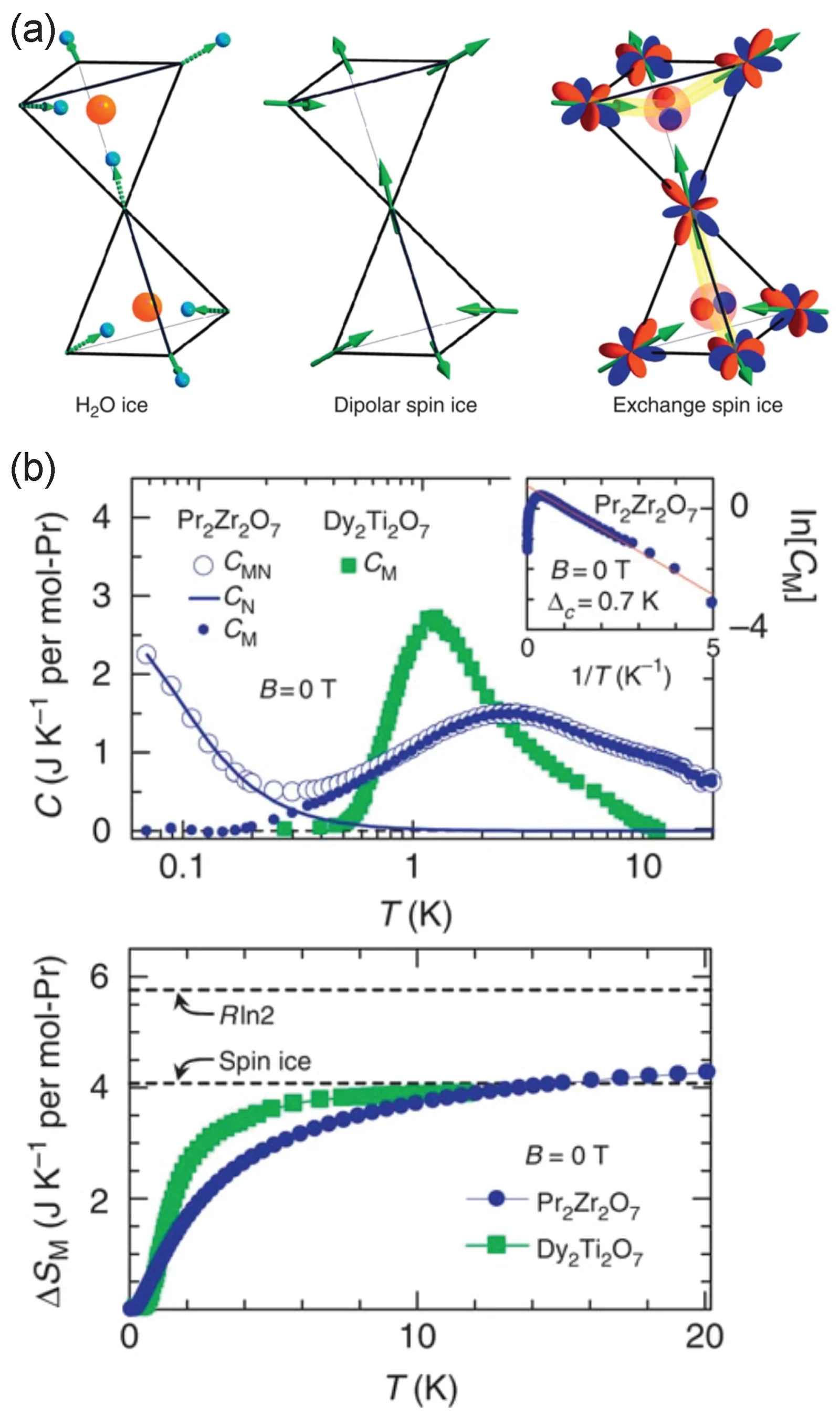
Ice-rule correspondence and thermodynamics of spin ice. (**a**) Schematic comparison of the local two-in–two-out constraint in water ice, classical dipolar spin ice, and exchange-based spin ice. Green arrows indicate local proton or magnetic-moment directions; red/blue lobes represent rare-earth 4f orbital character, and yellow/orange lobes indicate oxygen-2p-mediated exchange pathways. In water ice, the constraint concerns proton positions around tetrahedrally coordinated oxygen sites; in dipolar spin ice, it concerns rare-earth Ising moments along the local 〈111〉 axes; and the exchange-spin-ice schematic additionally shows rare-earth 4f orbital character and an oxygen-2p-mediated superexchange pathway. (**b**) Zero-field magnetic specific heat and magnetic entropy recovered upon warming in ${\mathrm{Pr}}_{2}$${\mathrm{Zr}}_{2}$$O_{7}$, compared with classical ${\mathrm{Dy}}_{2}$${\mathrm{Ti}}_{2}$$O_{7}$. The broad heat capacity response and the entropy recovered by integrating $C_{M}/T$ illustrate the thermodynamic consequences of the ice-rule manifold; the dashed lines mark the full Ising entropy Rln2 and the entropy release level expected when the Pauling residual entropy remains, Rln2−(R/2)ln(3/2). Adapted from Ref. [33]; the ${\mathrm{Dy}}_{2}$${\mathrm{Ti}}_{2}$$O_{7}$ thermodynamic data shown in panel (**b**) were originally reported in Ref. [24].

The concept of residual entropy was first established in ordinary water ice, illustrated in Figure 1a. In crystalline ice-I*h*, each oxygen atom is tetrahedrally coordinated by four neighboring oxygen atoms through hydrogen bonds [23]. Each proton lies along an O–O bond and can occupy a position closer to either neighboring oxygen. The lowest-energy configurations satisfy the ice rule: two protons lie close to each oxygen and two lie farther away [23]. This local constraint does not uniquely determine the global proton arrangement, leaving an exponentially large number of allowed configurations and hence a finite residual entropy.

Using a simple combinatorial argument, Pauling estimated the number of allowed proton configurations and showed that water ice retains a finite residual entropy [23],

(1) $$S_{0,\mathrm{ice}}=R\ln\left(\frac{3}{2}\right),$$

per mole of water molecules, even at zero temperature. This remarkable result established that local constraints alone can generate extensive configurational entropy without requiring chemical disorder, providing the conceptual foundation for the later discovery of spin ice.

### 2.2. Spin Ice: Crystal Structure and Magnetic Interactions

The magnetic analogue of water ice was discovered in rare-earth pyrochlore oxides with chemical formula $R_{2}M_{2}O_{7}$, where *R* is a magnetic rare-earth ion such as Dy or Ho and *M* is a nonmagnetic transition-metal ion, typically Ti or Sn [11,19,34]. The canonical compounds ${\mathrm{Dy}}_{2}$${\mathrm{Ti}}_{2}$$O_{7}$ and ${\mathrm{Ho}}_{2}$${\mathrm{Ti}}_{2}$$O_{7}$ crystallize in the cubic pyrochlore structure ($Fd\bar{3}m$), in which the magnetic rare-earth ions form a three-dimensional network of corner-sharing tetrahedra [18]. Strong crystal electric field effects constrain each rare-earth moment to point along its local 〈111〉 axis, producing an effective Ising spin that points either toward or away from the center of each tetrahedron. The middle schematic in Figure 1a illustrates this classical dipolar spin-ice limit and its direct correspondence with the local ice rule of water. The magnetic Hamiltonian contains nearest-neighbor exchange and long-range dipolar interactions [17],

(2) $$H=-J\sum_{\langle ij\rangle }S_{i}\cdot S_{j}+Dr_{\mathrm{nn}}^{3}\sum_{i<j}\frac{S_{i}\cdot S_{j}-3\left(S_{i}\cdot {\hat{r}}_{ij}\right)\left(S_{j}\cdot {\hat{r}}_{ij}\right)}{r_{ij}^{3}},$$

where *J* and *D* denote the exchange and dipolar coupling strengths, respectively. Owing to the exceptionally large magnetic moments (∼10 $\mu_{B}$) of ${\mathrm{Dy}}^{3+}$ and ${\mathrm{Ho}}^{3+}$, dipolar interactions play a crucial role in determining the low-energy magnetic properties and stabilizing the spin-ice state. The combined effects of local Ising anisotropy and magnetic interactions favor the 2I2O configuration on every tetrahedron, directly realizing the magnetic analogue of the water-ice rule shown in Figure 1a. Because this is a local constraint rather than a unique global spin arrangement, a macroscopic number of configurations remain accessible within the spin-ice manifold. For spin ice, Pauling counting gives the residual entropy $S_{0,\mathrm{SI}}=\left(R/2\right)\ln\left(3/2\right)$ per mole of magnetic ions [19,24]. For *N* Ising moments, the pyrochlore lattice contains N/2 tetrahedra, and the independent-tetrahedron Pauling estimate gives $W≃{\left(3/2\right)}^{N/2}$; this directly produces the factor of one-half relative to Equation (1). This entropy reflects correlated configurational degeneracy rather than random disorder and underlies the Coulomb-phase correlations and fractionalized excitations discussed below.

### 2.3. Calorimetric Observation of Residual Entropy

The residual entropy of spin ice was first established experimentally through calorimetric measurements on ${\mathrm{Dy}}_{2}$${\mathrm{Ti}}_{2}$$O_{7}$ [24]. The magnetic entropy released upon warming can be obtained by integrating the magnetic contribution to the specific heat,

(3) $$S_{\mathrm{mag}}\left(T\right)=\int_{0}^{T}\frac{C_{\mathrm{mag}}\left(T^{\prime }\right)}{T^{\prime }}\;dT^{\prime },$$

where $C_{\mathrm{mag}}$ is obtained after subtracting nonmagnetic contributions from the measured specific heat. At temperatures well above the spin-ice interaction scale, but still below the relevant crystal field excitations, the ice-rule correlations are thermally destroyed and each moment effectively samples its two local Ising states independently. The accessible spin configurations then approach $2^{N}$ for *N* Ising moments, corresponding to the full magnetic entropy Rln2 per mole of magnetic ions. Thus, the relevant high-temperature limit is set by the spin-ice energy scale, of order 1 K, rather than by an absolute temperature such as room temperature.

The classical benchmark for the thermodynamics in Figure 1b is the calorimetric observation of the Pauling entropy in ${\mathrm{Dy}}_{2}$${\mathrm{Ti}}_{2}$$O_{7}$ by Ramirez et al. [24], subsequently placed in the broader spin-ice framework by Bramwell and Gingras [19]. Kimura et al. later compared this classical response with the zero-field thermodynamics of the exchange-based spin-ice compound ${\mathrm{Pr}}_{2}$${\mathrm{Zr}}_{2}$$O_{7}$ [33]. For ${\mathrm{Dy}}_{2}$${\mathrm{Ti}}_{2}$$O_{7}$, the broad magnetic specific-heat anomaly is associated with the development of spin-ice correlations. Integrating $C_{\mathrm{mag}}/T$ yields an entropy release that approaches $R\ln2-S_{0,\mathrm{SI}}$, leaving the Pauling entropy unreleased in the experimentally accessible spin-ice regime [24]. This calorimetric deficit provided the original thermodynamic evidence for the macroscopically degenerate ice-rule manifold.

${\mathrm{Pr}}_{2}$${\mathrm{Zr}}_{2}$$O_{7}$ shows a broader magnetic specific-heat response, while its integrated magnetic entropy approaches the same classical spin-ice entropy release level by approximately 20 K [33]. The similarity of the recovered entropy emphasizes that the ice-rule constraint remains thermodynamically important even when quantum dynamics strongly modifies the low-energy response. Field-dependent calorimetry in ${\mathrm{Dy}}_{2}$${\mathrm{Ti}}_{2}$$O_{7}$ provides a complementary classical test: an applied magnetic field lifts the ice-rule degeneracy and drives the recovered entropy toward the full Rln2 value [24].

An important qualification is that the Pauling entropy characterizes the experimentally accessible spin-ice regime rather than proving that the macroscopic degeneracy must persist to the exact equilibrium ground state. Long-range dipolar interactions and weaker exchange terms can lift the degeneracy at sufficiently low temperature, while the rapid growth of spin relaxation times makes equilibration increasingly difficult. The relationship among the ideal dipolar spin-ice ground state, disorder, and the experimentally observed low-temperature freezing therefore remains an active problem [35,36,37].

### 2.4. Quantum Spin Ice

Quantum spin ice (QSI) extends the classical spin-ice problem by introducing coherent quantum dynamics into the ice-rule manifold. In classical compounds such as ${\mathrm{Dy}}_{2}$${\mathrm{Ti}}_{2}$$O_{7}$ and ${\mathrm{Ho}}_{2}$${\mathrm{Ti}}_{2}$$O_{7}$, strong crystal electric field anisotropy constrains the large rare-earth moments to the local 〈111〉 axes, so the low-energy degrees of freedom are well approximated as classical Ising variables and transitions between ice configurations occur predominantly through thermally activated spin flips [19,38]. In QSI candidates, the crystal field ground-state doublet instead supports appreciable transverse exchange. Representative systems include Pr-based non-Kramers pyrochlores and Ce-based dipole–octupole pyrochlores such as ${\mathrm{Pr}}_{2}$${\mathrm{Zr}}_{2}$$O_{7}$, ${\mathrm{Pr}}_{2}$${\mathrm{Hf}}_{2}$$O_{7}$, ${\mathrm{Ce}}_{2}$${\mathrm{Zr}}_{2}$$O_{7}$, and ${\mathrm{Ce}}_{2}$${\mathrm{Sn}}_{2}$$O_{7}$ [15,33,39,40,41,42,43,44,45,46]. Thus, whether a material behaves classically or quantum mechanically is determined not simply by the rare-earth element but by the crystal field doublet and the resulting exchange interactions.

The distinction can be seen directly from a minimal pseudospin-1/2 Hamiltonian,

(4) $$H_{\mathrm{QSI}}=J_{zz}\sum_{\langle ij\rangle }S_{i}^{z}S_{j}^{z}-J_{\pm }\sum_{\langle ij\rangle }\left(S_{i}^{+}S_{j}^{-}+S_{i}^{-}S_{j}^{+}\right)+\cdots ,$$

where *z* is the local 〈111〉 direction and the omitted terms represent additional symmetry-allowed anisotropic exchanges [15,38,47,48,49,50]. When the Ising interaction $J_{zz}>0$ dominates and the transverse terms are negligible, the Hamiltonian favors the macroscopically degenerate 2I2O manifold of classical spin ice. The key quantum ingredient is the finite transverse exchange $J_{\pm }$. Because the operators $S_{i}^{\pm }$ do not commute with $S_{i}^{z}$, they can coherently change the local Ising configuration. Through sequences of virtual spin flips, the system can tunnel between different 2I2O configurations without permanently leaving the ice-rule manifold. The ground state may therefore become a coherent superposition of many classical ice configurations rather than a frozen or thermally sampled ensemble. At long wavelengths, this quantum Coulomb phase is described by an emergent compact U(1) gauge theory supporting fractionalized matter excitations and a gapless photon-like collective mode [38,48,51,52].

Experimentally, the central challenge is to distinguish QSI from a disordered paramagnet or a slowly fluctuating classical spin ice. Neutron scattering is particularly useful because quantum fractionalization can generate broad continua and characteristic low-energy correlations rather than conventional sharp spin-wave modes. In ${\mathrm{Pr}}_{2}$${\mathrm{Hf}}_{2}$$O_{7}$, bulk measurements and neutron spectroscopy first established it as a QSI candidate and subsequently revealed spin-ice correlations together with an inelastic continuum consistent with quantum dynamics within the constrained manifold [39,40]. Ce-based dipole–octupole pyrochlores have since provided a particularly active experimental platform. In ${\mathrm{Ce}}_{2}$${\mathrm{Zr}}_{2}$$O_{7}$, neutron scattering, thermodynamic measurements, and microscopic modeling support a dynamic QSI regime and a candidate $U{\left(1\right)}_{\pi }$ quantum spin liquid [41,42,43], while recent diffuse scattering measurements further constrain the balance between dipolar and octupolar correlations [46]. ${\mathrm{Ce}}_{2}$${\mathrm{Sn}}_{2}$$O_{7}$ exhibits a gapped spectrum interpreted in terms of fractional matter coupled to an emergent gauge field [44], whereas ${\mathrm{Ce}}_{2}$${\mathrm{Zr}}_{2}$$O_{7}$ also shows low-energy magnetic excitations and thermodynamic behavior consistent with gapless emergent modes [45]. Complementary calculations of the field response of dipolar–octupolar QSI provide experimentally testable signatures for distinguishing candidate quantum spin-liquid regimes [53]. Although the detailed microscopic Hamiltonian remains material dependent, these developments substantially strengthen and sharpen the experimental case for a quantum analogue of the classical spin-ice Coulomb phase.

Entropy provides a useful distinction between the classical and quantum limits. Classical spin ice retains the Pauling configurational entropy because an exponentially large number of 2I2O states remain effectively degenerate over the accessible spin-ice regime. In an ideal QSI, transverse exchange coherently mixes and ultimately lifts this classical degeneracy, so an extensive Pauling residual entropy is not expected to persist as T→0. Instead, entropy is progressively released as quantum correlations develop, while the low-energy emergent excitations provide additional temperature-dependent contributions to the heat capacity and entropy [45,52,54]. QSI therefore preserves the frustrated ice manifold but transforms its role: the classical configurational degeneracy becomes the basis for a quantum-entangled state with dynamical emergent electrodynamics.

### 2.5. Artificial Spin Ice

**Figure 2:**
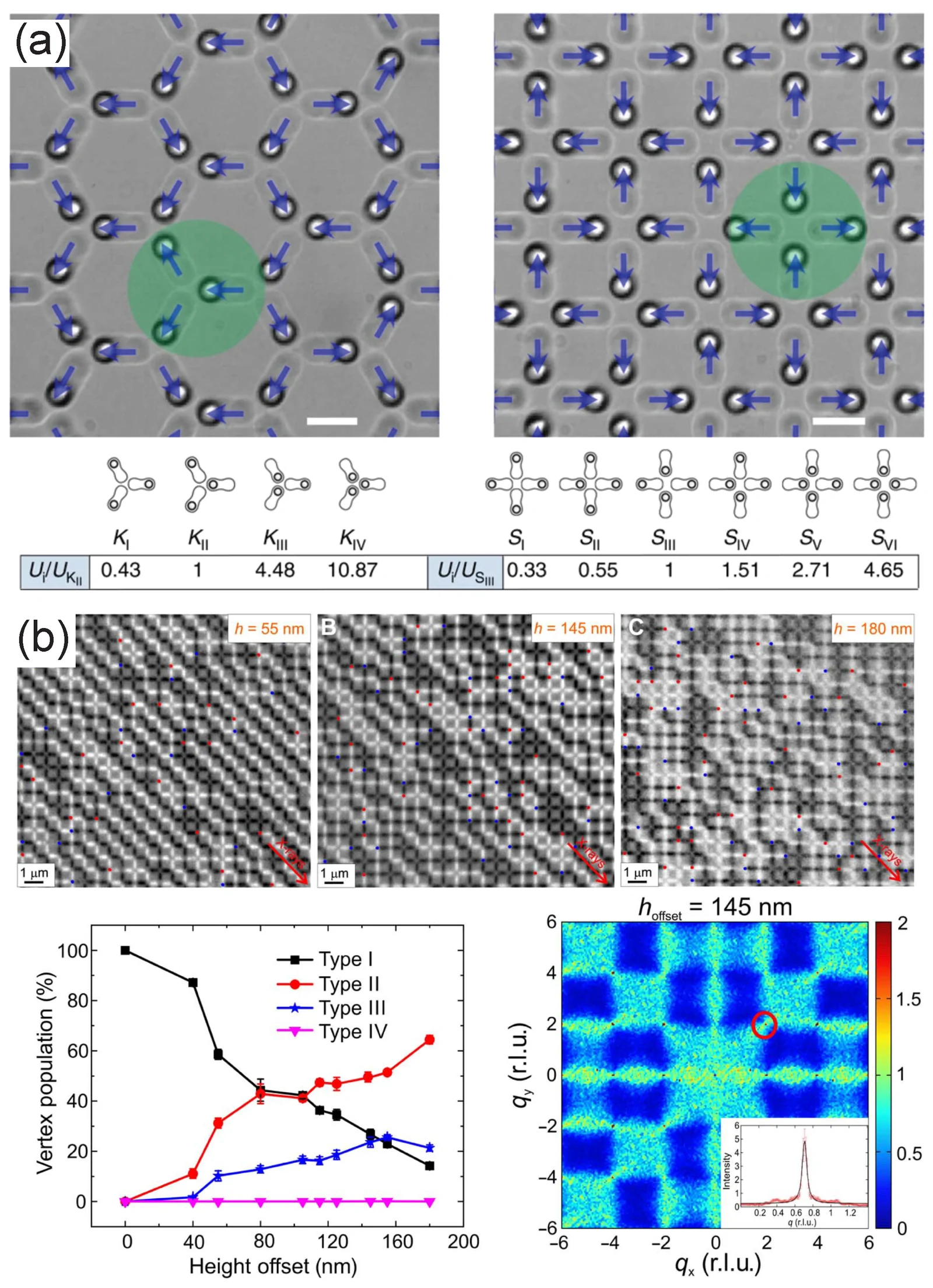
Artificial ice platforms and geometrically engineered degeneracy. (**a**) Experimental honeycomb and square colloidal artificial ice configurations formed by paramagnetic particles confined to bistable traps. Blue arrows denote the corresponding effective Ising directions, and the schematics below show the vertex classes together with their normalized magnetostatic energies. (**b**) Experimental tuning of nanomagnetic artificial square ice by introducing a vertical height offset between the two nanomagnet sublattices. Real-space magnetic configurations are shown for several offsets, together with the corresponding vertex populations. Near the critical offset around h=145–155 nm, the type-II population approaches twice the type-I population, as expected when the two ice-rule vertex classes become energetically degenerate because type II has twice the configurational multiplicity. The magnetic structure factor at h=145 nm exhibits pinch-point features characteristic of a Coulomb phase. The red circle marks the pinch point at $\left(q_{x},q_{y}\right)=\left(2,2\right)$, used for the line-scan analysis shown in the inset. Panel (**a**) is adapted from Ref. [56]; panel (**b**) is adapted from Ref. [57].

Artificial spin ice (ASI) takes a complementary route: instead of relying on atomic moments in a naturally occurring frustrated crystal, it constructs the ice problem from lithographically patterned ferromagnetic nanomagnets [6,8,55]. A typical ASI is fabricated by electron beam lithography and thin-film deposition as an array of elongated, single-domain islands arranged on square, kagome, honeycomb, or more elaborate networks. Shape anisotropy forces the magnetization of each island to lie approximately along its long axis, so every island acts as a mesoscopic Ising “macrospin.” Because the island dimensions, spacing, orientation, material, and even relative height can be chosen during fabrication, ASI turns the fixed atomic geometry of a bulk spin ice into a programmable magnetic interaction network. Nanomagnetic square and kagome arrays remain the canonical fabricated-spin realizations, as reviewed comprehensively by Skjærvø et al. [55]. Related ice-rule physics can also be realized in colloidal artificial ice, where paramagnetic particles confined to bistable traps define effective Ising variables. Figure 2a highlights this complementary platform through the honeycomb and square colloidal-ice configurations of Ortiz-Ambriz and Tierno, together with their vertex classes and relative energies [56].

The similarity to classical spin ice comes from the local ice rule. In square ASI, four islands terminate at each vertex, and the lowest-charge configurations contain two moments pointing into and two pointing out of the vertex. A convenient dumbbell description replaces each island by opposite magnetic charges ±q at its ends. The net charge of a vertex can then be written as [8,58]

(5) $$Q_{v}=q\left(N_{\mathrm{in}}-N_{\mathrm{out}}\right),$$

so a 2I2O vertex has $Q_{v}=0$, while 3I1O and 1I3O vertices carry opposite nonzero charges. Successive macrospin reversals move these charged defects and leave chains of reversed islands analogous to Dirac strings. The correspondence with atomic spin ice is not exact, however. In a conventional planar square ASI, the interaction between perpendicular nearest-neighbor islands differs from that between collinear opposing islands, so the six 2I2O configurations split into the lower-energy twofold type-I class and the fourfold type-II class; the true ground state is therefore ordered rather than extensively degenerate. Geometric design can restore the spin-ice-like degeneracy: vertically offsetting the two sublattices makes the relevant interactions nearly equivalent and produces an artificial square ice with an extensively degenerate manifold, Coulomb-phase correlations, and monopole-like excitations [57,59]. This tuning is illustrated in Figure 2b. As the height offset is increased, the vertex populations evolve away from the type-I ordered state; around h=145–155 nm, type-II vertices occur at approximately twice the population of type-I vertices, consistent with restoration of the degeneracy between the two ice-rule vertex classes after accounting for their multiplicities. At h=145 nm, the corresponding magnetic structure factor develops pinch-point features, providing a reciprocal-space signature of the emergent Coulomb phase [57]. More broadly, vertex-frustrated architectures such as shakti and tetris lattices demonstrate that artificial design can generate emergent ice rules, magnetic charge screening, and reduced-dimensional behavior with no direct counterpart in a natural spin-ice crystal [60,61]. ASI therefore provides a controlled way to separate the consequences of the local ice rule from those of the precise interaction geometry.

A defining advantage of ASI is that the microscopic degrees of freedom can be visualized directly. The original artificial square-ice experiments used magnetic force microscopy (MFM) to determine the orientation of individual islands and reconstruct the full microstate [6]. Synchrotron X-ray magnetic circular dichroism photoemission electron microscopy (XMCD-PEEM) later enabled real-space observation of monopole–antimonopole defects and their associated Dirac strings in kagome ASI [58]. Other implementations use Lorentz transmission electron microscopy (LTEM), electron holography, and related magnetic-imaging techniques [55]. Because each macrospin is a fabricated object rather than an atomic degree of freedom, the state can also be manipulated in unusually direct ways. Global rotating or oscillating magnetic fields can reconfigure entire arrays; thermally active islands can reverse spontaneously; and local defects, field sources, or deliberately modified island shapes can nucleate and guide selected charge excitations. Controlled defect sites have, for example, been used to inject and manipulate monopole-like charges through artificial spin-ice networks under applied fields [62]. Thermal protocols have enabled magnetic charge crystallization and direct real-space imaging of ordering transitions [63,64], while more recent clocking protocols provide stepwise control over collective domain dynamics [65].

This microscopic visibility also changes how entropy can be studied. In bulk spin ice, the configurational entropy is normally inferred indirectly from calorimetry through $\int C_{\mathrm{mag}}/T\;dT$. In ASI, a sufficiently large set of imaged microstates provides direct access to the probabilities of local configurations and therefore to configurational entropy. Lammert et al. developed a direct entropy-determination method for ASI and showed how correlations reduce the entropy below that of an uncorrelated Ising array [66]. An important qualification is that many early ASI arrays were effectively athermal during measurement because the reversal barrier of each island greatly exceeded $k_{B}T$; a field-demagnetized pattern therefore did not necessarily represent a thermal equilibrium ensemble. Thermally active arrays and fabrication protocols that permit equilibration have subsequently enabled direct studies of ground-state ordering and elementary excitations [55,67]. Recent experiments go further by demonstrating entropy-driven ordering in tetris ASI and directly imaging temperature-dependent ergodicity breaking, making entropy and equilibration themselves experimentally accessible observables [68,69].

From the perspective of this review, ASI is especially valuable because entropy can be engineered through geometry. Changing coordination number, island spacing, vertical offset, vacancies, edge termination, or interaction hierarchy changes the number and relative energies of accessible microstates. Classical pyrochlores therefore provide the microscopic equilibrium realization of the ice rule, QSI shows how quantum tunneling coherently mixes the constrained manifold, and ASI provides a platform in which that manifold can be fabricated, imaged, and deliberately reshaped. Three-dimensional ASI architectures extend this programmability toward diamond-bond networks in which mobile magnetic charges, tension-free Dirac strings, Coulomb-phase correlations, and competing charge-ordered phases can be explored directly [70,71,72]. This makes artificial spin ice a particularly transparent laboratory for studying entropy not only as a thermodynamic consequence of frustration, but also as a controllable design parameter for monopole-like excitations and collective magnetic states.

## 3. Pyrochlore Iridates and Entropic Phases

Pyrochlore iridates extend the spin-ice problem by introducing a second magnetic sublattice with strong spin-orbit coupling [14]. The ${\mathrm{Ir}}^{4+}$ moments order at temperatures far above the rare-earth spin-ice scale and, through the *d*–*f* exchange interaction, generate an effective local field on the rare-earth moments. Competition between this field and the intrinsic spin-ice interaction produces 2I2O, fragmented, and AIAO states [21,25], together with finite-temperature phases in which entropy is enhanced by competition between neighboring magnetic orders.

### 3.1. Crystal Structure, Magnetic Interactions, and Ground States

**Figure 3:**
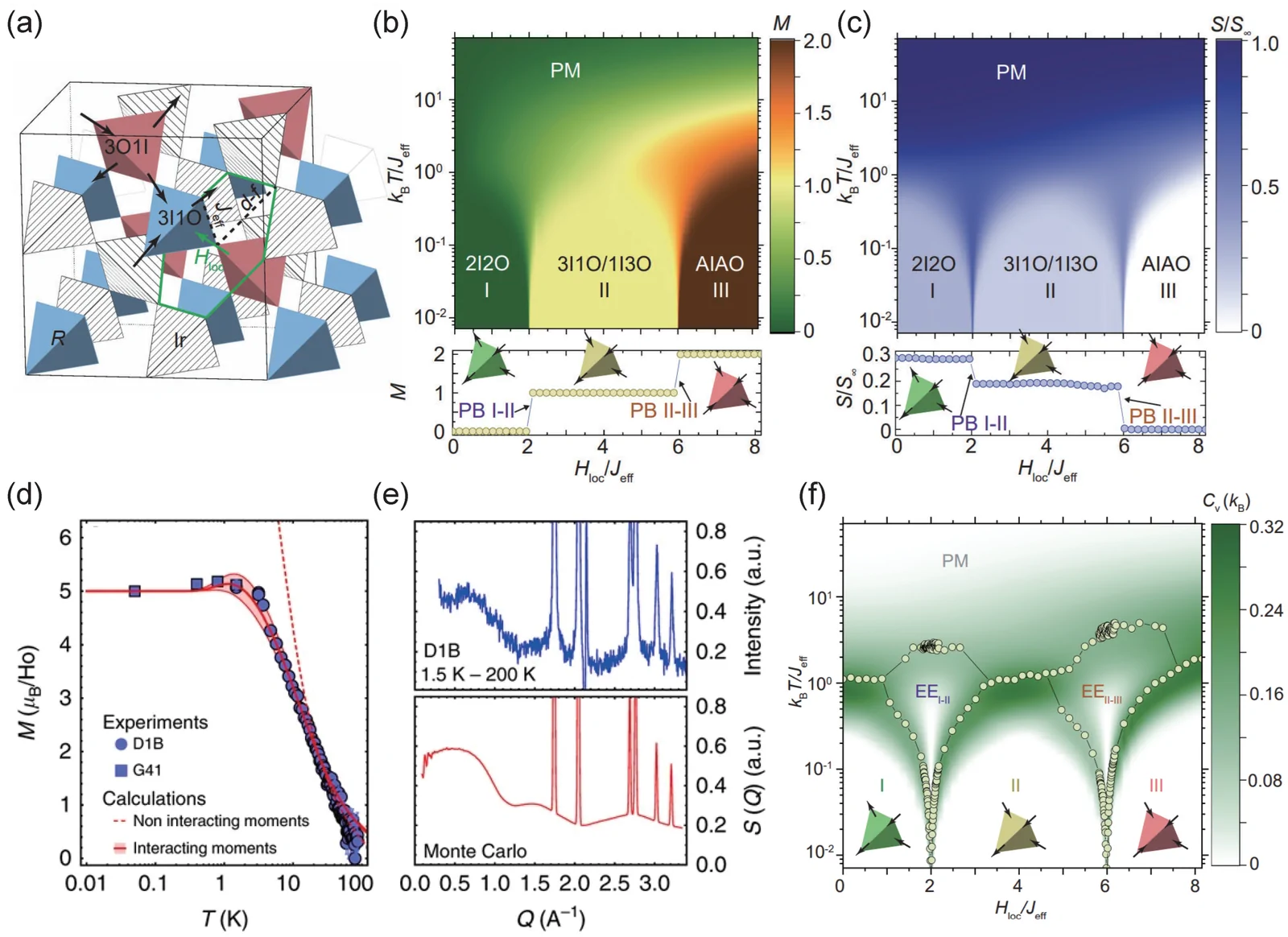
Magnetic phases and entropy enhancement in pyrochlore iridates. (**a**) Interpenetrating rare-earth and Ir pyrochlore sublattices and representative rare-earth spin configurations. Black arrows indicate the local rare-earth Ising moment directions along the 〈111〉 axes, while the green arrow denotes the Ir-induced local field $H_{\mathrm{loc}}$. The ordered Ir moments generate an effective local field on the rare-earth moments through the *d*–*f* exchange interaction. (**b**) Calculated rare-earth magnetic phase diagram as a function of temperature and $H_{\mathrm{loc}}/J_{\mathrm{eff}}$, showing the 2I2O (I), fragmented (II), AIAO (III), and high-temperature paramagnetic (PM) phases. (**c**) Corresponding entropy map, illustrating the distinct residual entropies of the low-temperature phases. (**d**) Temperature dependence of the ordered ${\mathrm{Ho}}^{3+}$ moment in ${\mathrm{Ho}}_{2}$${\mathrm{Ir}}_{2}$$O_{7}$ measured by neutron diffraction and compared with Monte Carlo simulations. (**e**) Experimental and calculated magnetic diffuse scattering, demonstrating the coexistence of long-range order and a fluctuating component in the fragmented phase. (**f**) Calculated heat capacity map showing two finite-temperature entropy-enhanced (EE) phases near the I–II and II–III ground-state boundaries. Adapted from Refs. [21,25].

Pyrochlore iridates have the chemical formula $R_{2}$${\mathrm{Ir}}_{2}$$O_{7}$, where *R* is a trivalent rare-earth ion [14,18]. The rare-earth and Ir ions form independent, interpenetrating networks of corner-sharing tetrahedra, as illustrated in Figure 3a. For spin-ice rare-earth ions such as Ho and Dy, the rare-earth moments retain strong local 〈111〉 Ising anisotropy, whereas the Ir 5d electrons experience strong spin-orbit coupling and electronic correlations that stabilize AIAO order over a broad range of pyrochlore iridates.

A complementary experimental perspective is provided by ${\mathrm{Er}}_{2}$${\mathrm{Ir}}_{2}$$O_{7}$. Ezairi et al. confirmed the cubic $Fd\bar{3}m$ pyrochlore structure by X-ray diffraction and reported a weak ferromagnetic transition near 140 K, which they attributed to the onset of magnetic order of the Ir moments [26]. Their magnetocaloric analysis quantified the field-induced magnetic entropy change and yielded relative cooling powers of 98 and 128 J ${\mathrm{kg}}^{-1}$ for field changes of 40 and 50 kOe, respectively. The temperature-averaged entropy change remained close to the peak magnetic entropy change, which the authors interpreted as indicating relatively small hysteresis losses. This compound-specific magnetocaloric result provides a complementary thermodynamic perspective to the AIAO-based framework discussed below.

Unlike the rare-earth moments, the Ir 5d moments order into a long-range AIAO antiferromagnetic state at a much higher temperature, typically around 100–150 K [21,73]. Because this ordering temperature is nearly two orders of magnitude larger than the characteristic energy scale of the rare-earth moments (∼1 K), the Ir magnetic structure is already well established when the rare-earth spins begin to develop spin-ice correlations. The AIAO state does not possess the extensive degeneracy of spin ice. Because the Ir order is already established on the temperature scale relevant to the rare-earth moments, it is therefore convenient to treat the Ir sublattice as a static magnetic background that acts on the rare-earth moments through the *d*–*f* exchange interaction.

Under this approximation, the rare-earth magnetic state can be described by the effective Hamiltonian [21]

(6) $$H=J_{\mathrm{eff}}\sum_{\langle ij\rangle }\sigma_{i}\sigma_{j}-H_{\mathrm{loc}}\sum_{i}\sigma_{i},$$

where $\sigma_{i}=\pm 1$ denotes the Ising moment along the local 〈111〉 direction. The first term, characterized by the effective nearest-neighbor interaction $J_{\mathrm{eff}}$, is the conventional spin-ice interaction that favors the 2I2O configuration on each tetrahedron. The second term represents the effective local field $H_{\mathrm{loc}}$ generated by the ordered Ir moments through the *d*–*f* exchange interaction. This field favors the AIAO configuration of the rare-earth moments. Consequently, the magnetic ground state of the rare-earth sublattice is determined by the competition between the spin-ice interaction $J_{\mathrm{eff}}$ and the Ir-induced local field $H_{\mathrm{loc}}$, with the ratio $H_{\mathrm{loc}}/J_{\mathrm{eff}}$ serving as the primary tuning parameter of the phase diagram described by Equation (6).

The calculated magnetic phase diagram is shown in Figure 3b. At high temperatures, thermal fluctuations overcome both the spin-ice interaction and the Ir-induced local field, resulting in the paramagnetic state with disordered rare-earth moments. Upon cooling, the magnetic ground state is determined by the ratio $H_{\mathrm{loc}}/J_{\mathrm{eff}}$. For $H_{\mathrm{loc}}/J_{\mathrm{eff}}<2$, the effective spin-ice interaction dominates, stabilizing the conventional 2I2O spin-ice state (Phase I). In the intermediate regime, $2<H_{\mathrm{loc}}/J_{\mathrm{eff}}<6$, neither interaction is sufficiently strong to completely suppress the other, giving rise to a fragmented magnetic state (Phase II). This phase consists of three-in–one-out (3I1O) and one-in–three-out (1I3O) tetrahedra and combines long-range magnetic order with a macroscopically degenerate fluctuating component. When $H_{\mathrm{loc}}/J_{\mathrm{eff}}>6$, the *d*–*f* exchange interaction dominates, forcing all rare-earth moments into the AIAO antiferromagnetic configuration (Phase III). Thus, increasing $H_{\mathrm{loc}}/J_{\mathrm{eff}}$ drives the rare-earth sublattice from the 2I2O spin-ice state to AIAO order through the intermediate fragmented phase.

The entropy evolution corresponding to the magnetic phase diagram is shown in Figure 3c. In the high-temperature paramagnetic phase, thermal fluctuations completely overcome the spin interactions, allowing each rare-earth moment to freely occupy either of its two local Ising states. Consequently, the magnetic entropy reaches the full Ising value of Rln2. Upon cooling, the residual entropy depends on the competition between the spin-ice interaction and the Ir-induced local field. In the spin-ice regime (Phase I), the system retains the Pauling residual entropy $S_{0,\mathrm{SI}}\approx \left(R/2\right)\ln\left(1.5\right)$ discussed above, reflecting the macroscopic degeneracy of the 2I2O manifold. In contrast, the AIAO phase (Phase III) has no extensive configurational degeneracy and therefore carries no residual entropy in the thermodynamic limit.

The most remarkable behavior occurs in the intermediate fragmented phase (Phase II), where the residual entropy is reduced to approximately $S_{\mathrm{frag}}\approx \left(R/2\right)\ln\left(1.3\right)$ [21,74]. Unlike conventional magnetic ordering, the fragmented state simultaneously exhibits long-range magnetic order and a fluctuating spin-ice background. As a result, the Ir-induced local field lifts only part of the spin-ice degeneracy, while a large number of spin configurations remain accessible. This intermediate residual entropy is a defining thermodynamic signature of magnetic fragmentation, distinguishing it from both the highly degenerate spin-ice state and the fully ordered AIAO phase.

The magnetic fragmentation scenario is strongly supported by neutron scattering experiments on ${\mathrm{Ho}}_{2}$${\mathrm{Ir}}_{2}$$O_{7}$ [21]. As shown in Figure 3d, the ordered Ho moment extracted from magnetic Bragg scattering grows upon cooling, demonstrating the development of long-range order on the rare-earth sublattice. Its temperature dependence is well reproduced by Monte Carlo simulations with $H_{\mathrm{loc}}/J_{\mathrm{eff}}\approx 4.5$, placing ${\mathrm{Ho}}_{2}$${\mathrm{Ir}}_{2}$$O_{7}$ within the fragmented regime. Remarkably, the ordered moment saturates at only about one-half of the full Ho Ising moment at low temperatures, indicating that only part of the magnetic moment contributes to the static AIAO order. At the same time, diffuse magnetic scattering persists, as shown in Figure 3e. The coexistence of diffuse scattering with AIAO Bragg peaks, together with the excellent agreement between experiment and Monte Carlo simulations, demonstrates that a fluctuating spin-ice component survives alongside the ordered magnetic component. These observations provide compelling experimental evidence for magnetic fragmentation in pyrochlore iridates.

### 3.2. Entropy-Enhanced Phases

The three ground states define the energetic backbone of the phase diagram, but the finite-temperature behavior is richer. At finite temperature, the competition between the spin-ice interaction and the Ir-induced local field produces a considerably richer structure. Near the two ground-state phase boundaries, where neighboring magnetic phases are nearly degenerate in energy, thermal fluctuations become particularly effective in exploring a large configurational phase space. As a result, entropy becomes an essential contribution to the free energy, giving rise to two distinct EE phases located between the spin-ice and fragmented phases (${\mathrm{EE}}_{I-\mathrm{II}}$) and between the fragmented and AIAO phases (${\mathrm{EE}}_{\mathrm{II}-\mathrm{III}}$), respectively [25].

The thermodynamic signatures of these states are summarized in Figure 3f. Away from the ground-state boundaries, the heat capacity exhibits a single dominant anomaly upon cooling. Near either the I–II or II–III boundary, however, this anomaly splits into two peaks, defining an additional finite-temperature regime between the paramagnetic state and the low-temperature phase. These regimes, denoted ${\mathrm{EE}}_{I-\mathrm{II}}$ and ${\mathrm{EE}}_{\mathrm{II}-\mathrm{III}}$, occur only where the competing magnetic configurations are close in energy. The split heat capacity structure therefore identifies finite-temperature states that are absent from the ground-state phase diagram and are stabilized by the enhanced configurational phase space near the phase boundaries.

The enhanced entropy of the EE phases can be understood directly from their mixed magnetic structures. At the I–II boundary, ${\mathrm{EE}}_{I-\mathrm{II}}$ consists of approximately 54% phase-I tetrahedra and 46% phase-II tetrahedra, with an entropy substantially larger than that of either neighboring phase. A simple entropy maximization picture captures the origin of this enhancement. If $r_{I}$ is the fraction of phase-I tetrahedra, the molar entropy per mole of rare-earth moments in the mixed I–II state can be approximated as

(7) $$S_{I-\mathrm{II}}=r_{I}s_{I}+\left(1-r_{I}\right)s_{\mathrm{II}}+\frac{R}{2}\left[-r_{I}\ln r_{I}-\left(1-r_{I}\right)\ln\left(1-r_{I}\right)\right],$$

where $s_{I}=\left(R/2\right)\ln\left(3/2\right)$ and $s_{\mathrm{II}}\approx \left(R/2\right)\ln\left(1.3\right)$ are the configurational entropies of phases I and II, respectively. The first two terms represent the entropy inherited from the two constituent magnetic phases, whereas the last term is the additional mixing entropy associated with distributing the two types of tetrahedra throughout the lattice. At the I–II boundary, where the two competing phases are energetically degenerate, maximizing $S_{I-\mathrm{II}}$ with respect to $r_{I}$ gives $r_{I}\approx 53.6\%$ and $r_{\mathrm{II}}\approx 46.4\%$, remarkably close to the approximately 54% and 46% fractions obtained directly from the Monte Carlo simulations. Maximizing Equation (7) predicts $S_{I-\mathrm{II}}\approx 0.74R\ln2$, while the simulations yield approximately (R/2)ln(2.6)≈0.69Rln2. The quantitative difference reflects geometric constraints arising because neighboring tetrahedra share common spins and therefore cannot be mixed completely independently. A similar mechanism operates at the II–III boundary, where ${\mathrm{EE}}_{\mathrm{II}-\mathrm{III}}$ contains approximately 64% phase-II and 36% phase-III tetrahedra and retains an entropy of approximately (R/2)ln(1.94), substantially exceeding that of the neighboring fragmented phase, while the AIAO phase has zero residual entropy. Thus, the large entropy of both EE phases originates from the combination of the configurational degeneracy of their constituent magnetic states and the additional freedom associated with mixing them. Near the phase boundaries, where the competing configurations have nearly identical internal energies, this additional entropy becomes thermodynamically decisive. At finite temperatures, the −TS contribution lowers the free energy of the mixed EE states sufficiently to overcome their small energetic disadvantage, allowing them to become stable over finite temperature ranges. The EE phases are therefore stabilized by entropy-driven free energy minimization rather than by internal-energy minimization alone. The present evidence for these EE phases is primarily theoretical and computational, and establishing their thermodynamic signatures experimentally remains an important next step [25].

## 4. Emergent Magnetic Monopoles in Spin Ice

The possibility of isolated magnetic charges has fascinated physicists for more than a century. In conventional Maxwell electrodynamics, magnetic fields satisfy ∇·B=0, reflecting the absence of magnetic charge. In 1931, Dirac showed that magnetic monopoles can nevertheless be incorporated consistently into quantum electrodynamics and that the existence of even a single monopole would explain the quantization of electric charge [75]. In Dirac’s construction, the monopole is attached to an unobservable singular flux line, now known as the Dirac string, and the requirement that this string remain physically invisible leads to the Dirac quantization condition. Despite extensive searches in particle physics and cosmology, no elementary magnetic monopole has been experimentally confirmed.

A condensed-matter realization emerged in spin ice [22]. Local violations of the ice rule behave as emergent magnetic charges that can separate, interact through an effective Coulomb potential, and remain connected by Dirac strings. These excitations are not fundamental particles, but they reproduce key elements of monopole electrodynamics within the low-energy description of the spin system. Their theoretical prediction established spin ice as a paradigmatic example of quasiparticle fractionalization, and subsequent neutron scattering, magnetization, susceptibility, and magnetic-noise experiments have provided complementary evidence for their charge, correlations, and dynamics [27,28,29].

### 4.1. Violation of the Ice Rule and Monopole Creation

**Figure 4:**
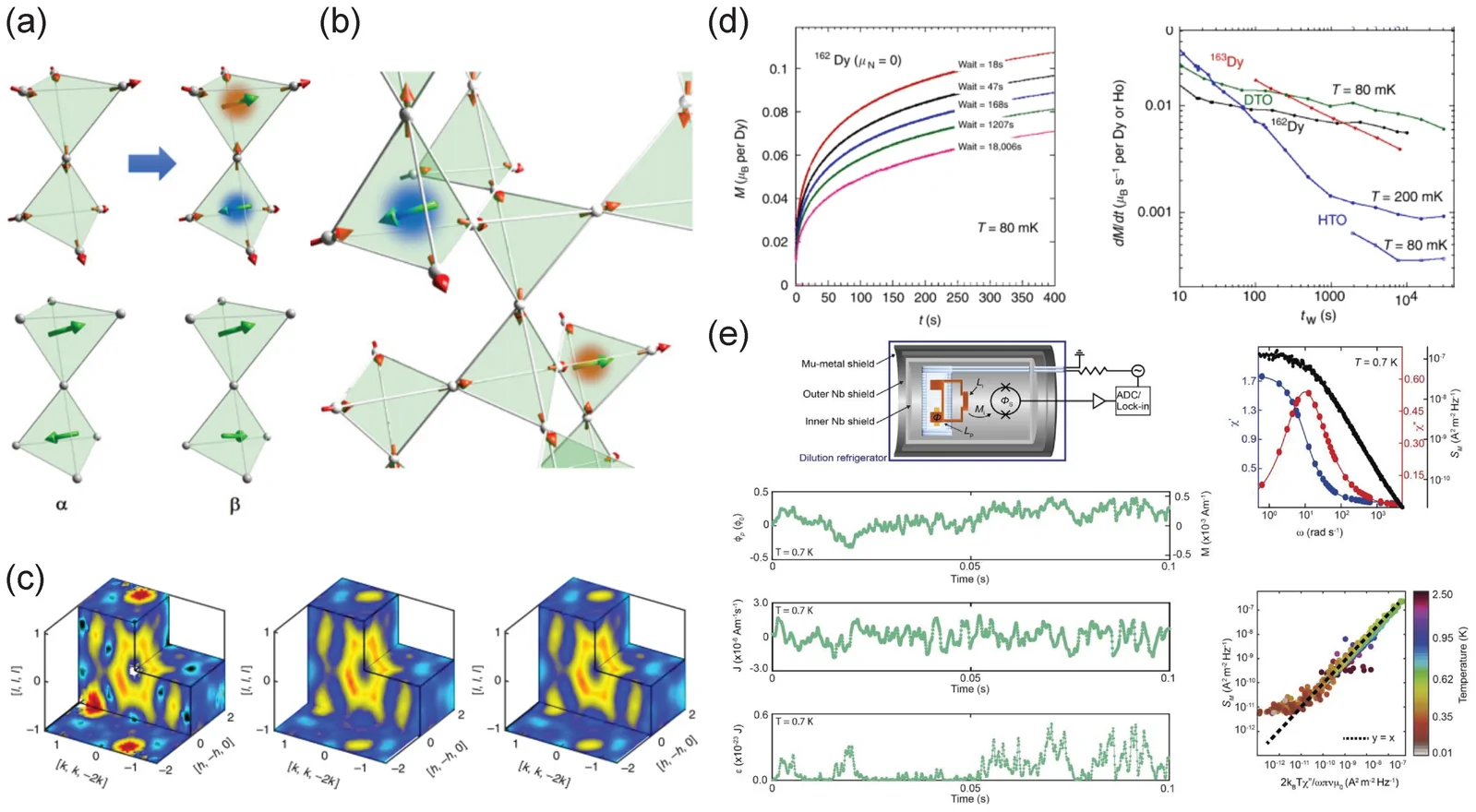
Emergent magnetic monopoles: microscopic configurations and experimental probes in spin ice. (**a**) Creation of a monopole–antimonopole pair by flipping the spin (green arrows) shared by two neighboring ice-rule tetrahedra. The resulting 3I1O and 1I3O defects (red and blue spheres) carry opposite effective magnetic charges and, in the microscopic model shown, are accompanied by local electric dipoles (green arrows); the lower schematics show the two relative dipole orientations, α and β, associated with monopole pair creation. (**b**) Spatially separated 1I3O and 3I1O monopoles in the pyrochlore network, indicated by oppositely colored monopole markers; the associated local electric dipoles are again shown by green arrows. (**c**) Three-dimensional diffuse neutron scattering from ${\mathrm{Dy}}_{2}$${\mathrm{Ti}}_{2}$$O_{7}$ at 680 mK: raw experimental scattering (left), the autoencoder-filtered experimental scattering (middle), and simulated scattering for the optimized spin Hamiltonian (right). (**d**) Nuclear spin dependence of monopole dynamics. Left: magnetization relaxation of ^162^Dy_2_${\mathrm{Ti}}_{2}$$O_{7}$ at 80 mK following application of a 0.08 T field after different zero-field wait times. Right: the initial monopole current $J_{m}\left(t=0\right)=dM/dt$ as a function of wait time for isotope-engineered ${\mathrm{Dy}}_{2}$${\mathrm{Ti}}_{2}$$O_{7}$ and ${\mathrm{Ho}}_{2}$${\mathrm{Ti}}_{2}$$O_{7}$. (**e**) SQUID-based magnetic-monopole noise spectrometry in ${\mathrm{Dy}}_{2}$${\mathrm{Ti}}_{2}$$O_{7}$, showing the experimental apparatus; representative time traces of pickup-coil flux, the derived monopole current $J={\dot{\Phi }}_{p}/\mu_{0}$, and magnetic energy at 700 mK; coterminous susceptibility and magnetization-noise spectra; and a fluctuation–dissipation comparison as a function of temperature. Panels (**a**,**b**) are adapted from Figure 1b,c and Figure 1a, respectively, of Ref. [76]; panel (**c**) from Figure 2a–c of Ref. [77]; panel (**d**) from Figure 3a,f of Ref. [78]; and panel (**e**) from Figure 1 of Ref. [79].

The low-energy excitations of spin ice are qualitatively different from the spin waves of a conventionally ordered magnet [22]. A local violation of the ice rule costs a finite excitation energy and is therefore thermally activated. As illustrated in Figure 4a, flipping a spin shared by two neighboring tetrahedra converts two 2I2O configurations into 3I1O and 1I3O defects. These defects carry opposite effective magnetic charges and form a monopole–antimonopole pair. In the microscopic representation reproduced in Figure 4a, each monopole is also accompanied by a local electric dipole; the α and β configurations denote two relative dipole orientations that split the monopole pair creation energy [76]. These electric dipoles are an additional microscopic degree of freedom and are not required for the magnetic charge definition of the monopole itself.

Once created, the pair can separate through successive spin flips [22,30]. Figure 4b illustrates the resulting spatially separated 1I3O and 3I1O monopoles embedded in the corner-sharing tetrahedral network [76]. After the initial pair-creation event, each subsequent flip transfers one charge to a neighboring tetrahedron while restoring the ice rule behind it. The original local spin flip therefore fractionalizes into two spatially separated quasiparticles carrying opposite magnetic charge. This deconfined motion is the central reason that the defects are described as emergent monopoles rather than localized spin excitations.

The long-range interaction between these quasiparticles originates from the dipolar interaction of the underlying Ising moments discussed in Section 2. Castelnovo et al. showed that the dipolar spin-ice Hamiltonian can be recast in terms of effective magnetic charges whose leading interaction is Coulombic [22],

(8) $$V\left(r\right)=\frac{\mu_{0}}{4\pi }\frac{Q_{1}Q_{2}}{r},$$

where $Q_{1}$ and $Q_{2}$ are the effective magnetic charges and *r* is their separation. As expressed by Equation (8), for a monopole–antimonopole pair $Q_{1}Q_{2}<0$, so the interaction is attractive. Unlike an elementary Dirac monopole, the magnetic charge and its Coulomb interaction are emergent properties of the collective spin configuration. Spin ice therefore realizes a magnetic Coulomb phase whose fractionalized excitations interact as effective magnetic charges.

### 4.2. Dirac Strings in Spin Ice

The concept of a Dirac string originates from Dirac’s theory of magnetic monopoles [75]. An isolated magnetic monopole produces a radial magnetic field with nonzero magnetic flux through a closed surface, apparently conflicting with the conventional condition ∇·B=0. Dirac showed that a monopole can nevertheless be consistently incorporated into electromagnetic theory if it is attached to an infinitely thin line carrying magnetic flux, now known as the Dirac string. The position of this string is gauge dependent and therefore cannot be physically observed; only its endpoint, the magnetic monopole, represents a physical excitation. The requirement that the Dirac string remain unobservable leads directly to the Dirac quantization condition for electric and magnetic charges.

A closely analogous structure emerges naturally in spin ice. Separating a monopole–antimonopole pair requires a sequence of spin flips, leaving behind a continuous chain of reversed spins connecting the two magnetic charges. This chain plays the role of a Dirac string: its endpoints correspond to the observable monopole and antimonopole, whereas the particular path connecting them is not uniquely defined. Different sequences of spin flips can connect the same two monopoles while remaining within the spin-ice manifold, and thermal fluctuations can continuously rearrange these paths. Thus, although the spin-ice Dirac string has a microscopic realization as a chain of reversed magnetic moments, its precise trajectory is not an independently conserved or uniquely identifiable object. The physically meaningful quantities are the monopole positions and the statistical correlations associated with the ensemble of allowed strings.

Polarized neutron scattering provided key experimental evidence for these string correlations [27]. Neutron scattering does not image an individual string; instead, it probes the collective spin correlations generated by the constrained spin-ice manifold. A complementary modern diffuse scattering comparison is shown in Figure 4c. Samarakoon et al. measured the three-dimensional diffuse scattering of ${\mathrm{Dy}}_{2}$${\mathrm{Ti}}_{2}$$O_{7}$ at 680 mK and compared the raw experimental data with an autoencoder-filtered representation and Monte Carlo scattering calculated for an optimized spin Hamiltonian [77]. The comparison shows how machine learning-assisted artifact removal, together with Monte Carlo modeling and thermodynamic constraints, can be used to quantitatively constrain the microscopic spin Hamiltonian underlying the diffuse correlations. The direct evidence for Dirac-string correlations remains the polarized neutron scattering work of Morris et al. [27].

### 4.3. Experimental Observation of Emergent Magnetic Monopoles

Following the theoretical prediction of emergent monopoles, experiments sought signatures that distinguish mobile magnetic charges from generic spin defects. A prominent early example is the magnetic analogue of the Wien effect in ${\mathrm{Dy}}_{2}$${\mathrm{Ti}}_{2}$$O_{7}$. An applied field favors the dissociation of bound monopole–antimonopole pairs and increases the population of free magnetic charges. From the field dependence of the magnetic response, Bramwell et al. extracted an effective monopole charge consistent with the spin-ice prediction and introduced the term “magnetricity” for magnetic charge transport [28]. This experiment provided a quantitative test of the monopole description by relating the microscopic spin-ice model to a response formulated in terms of mobile magnetic charge.

Figure 4d instead highlights a later transport experiment that probes the low-temperature monopole current. Paulsen et al. prepared spin-ice samples with different nuclear spin contents, allowed them to evolve for controlled zero-field wait times, and then measured the magnetization after applying a constant magnetic field [78]. The magnetization relaxation gives the magnetic current density $J_{m}=dM/dt$, and the right-hand plot shows its initial value $J_{m}\left(t=0\right)$ as a function of wait time. Because $J_{m}$ depends on monopole density, mobility, and susceptibility, these factors cannot be fully separated from a single relaxation trace; however, comparison among isotopically distinct samples reveals a systematic nuclear spin dependence and provides strong evidence that nuclear spin-assisted quantum tunnelling enhances monopole mobility in the low-temperature frozen regime [78].

Magnetic-flux-noise spectroscopy provides a complementary probe of monopole dynamics. The earlier SQUID measurements of Dusad et al. established characteristic equilibrium flux-noise spectra associated with thermally generated magnetic monopoles in ${\mathrm{Dy}}_{2}$${\mathrm{Ti}}_{2}$$O_{7}$ [29]. More recent SQUID-based measurements by Dasini et al. combine microsecond-resolved spontaneous magnetic-flux noise with coterminous magnetic-susceptibility measurements over a broad temperature range [79]. As summarized in Figure 4e, the superconducting pickup circuit measures the spontaneous pickup-coil flux $\Phi_{p}\left(t,T\right)$, with the SQUID output independently calibrated to the sample magnetization; differentiating the pickup flux gives the monopole-current component $J\left(t,T\right)={\dot{\Phi }}_{p}/\mu_{0}$. The same apparatus determines the magnetization-noise power spectrum $S_{M}\left(\omega ,T\right)$ together with $\chi^{\prime }\left(\omega ,T\right)$ and $\chi^{\prime \prime }\left(\omega ,T\right)$. At 700 mK the time- and frequency-domain data directly resolve monopole fluctuations. Comparing $S_{M}$ with the fluctuation–dissipation expectation shows the ergodicity parameter beginning to diminish near 500 mK and being manifestly lost by T≲250 mK. The time-resolved measurements further reveal intense monopole-current bursts in the supercooled regime, providing evidence for dynamical heterogeneity in the monopole fluid [79].

Other experiments have probed monopole motion away from equilibrium. Field-pulse measurements [31] in ${\mathrm{Dy}}_{2}$${\mathrm{Ti}}_{2}$$O_{7}$ generated long-lived magnetic currents that relaxed over macroscopic time scales, while frequency-dependent susceptibility measurements identified diffusive, Brownian-like monopole dynamics [31,32]. Together with polarized and diffuse neutron scattering [20,27,77], the magnetic Wien effect [28], isotope-dependent monopole transport [78], and magnetic-noise spectroscopy [29,79], these results establish a consistent quasiparticle picture in which magnetic charge possesses measurable interactions, transport, relaxation, and fluctuations across a wide range of time scales. This experimentally grounded monopole description provides the starting point for comparing how monopole-like and fractionalized excitations evolve in related spin-ice platforms.

### 4.4. Monopole Excitations Across Spin-Ice Platforms

The monopole concept extends beyond classical spin ice, but the microscopic meaning of the excitation changes from one platform to another. In classical spin ice, 3I1O and 1I3O tetrahedra are thermally activated violations of the 2I2O constraint and behave as mobile magnetic charges. In pyrochlore iridates, the staggered field generated by the ordered Ir sublattice can instead inject such charges into equilibrium: in the fragmented regime, 3I1O and 1I3O tetrahedra form an ordered monopole crystal coexisting with a fluctuating Coulomb-phase component, while low-energy charge defects can propagate through the resulting periodic background [21,74].

QSI promotes the static gauge structure of classical spin ice to a dynamical emergent U(1) gauge theory. Its excitation spectrum is therefore richer than a simple gas of classical monopoles. The theory contains gapped fractionalized gauge charges (spinons), a dual sector of gapped topological monopoles, and a gapless photon-like collective mode [38,51]. The identification of “electric” and “magnetic” gauge charges depends on the duality convention, so it is most useful here to regard the spinons as the quantum descendants of ice-rule-violating matter excitations and the emergent photon as a genuinely new collective excitation. Neutron scattering theory predicts suppression of the classical pinch-point singularities and a linearly dispersing photon mode [52]; recent spectroscopy on ${\mathrm{Ce}}_{2}$${\mathrm{Sn}}_{2}$$O_{7}$ and thermodynamic and neutron measurements on ${\mathrm{Ce}}_{2}$${\mathrm{Zr}}_{2}$$O_{7}$ provide evidence for fractionalized matter and emergent gauge excitations in candidate QSI materials [44,45].

ASI provides the most direct real-space realization of monopole-like excitations. Here the magnetic charge resides at vertices of lithographically patterned macrospin arrays rather than on atomic tetrahedra. Reversal of one or more islands creates oppositely charged vertex defects connected by strings of reversed islands. Such monopole–antimonopole pairs and their Dirac strings have been directly imaged in artificial kagome ice [58]; engineered square ice realizes local monopole analogues moving within a macroscopically degenerate Coulomb phase [59]; and thermally active or defect-engineered arrays permit real-time observation, injection, and controlled propagation of these charges [57,62]. Table 1 summarizes how the magnetic states, entropy, excitations, and experimental signatures compare across the four platforms.

In classical spin ice and fragmented pyrochlore iridates, the effective magnetic charge is carried by 3I1O and 1I3O tetrahedra formed from atomic Ising moments. QSI has a different excitation structure, with fractionalized gauge charges, topological monopoles, and an emergent photon, while ASI realizes charged mesoscopic vertices connected by strings of reversed islands. The 2DMG considered in the next section is built from the classical 3I1O/1I3O defects: the iridate boundary generates an excess of one charge sign and confines those monopoles near the interface.

## 5. Theoretically Proposed Two-Dimensional Magnetic Monopole Gas and Entropic Confinement

In bulk spin ice, monopoles are thermally created as monopole–antimonopole pairs [22], so the populations of positive and negative magnetic charge remain equal and the net charge vanishes. Within the low-temperature ice-rule manifold, the monopole density also decreases rapidly because pair creation costs a finite energy. Thus, although individual monopoles can move and interact, their charge density cannot be independently populated in the manner familiar from electronic carrier systems.

This limitation motivated the theoretical proposal of a two-dimensional magnetic monopole gas (2DMG) at an engineered spin-ice/pyrochlore iridate interface [80]. The 2DMG has not yet been experimentally realized; throughout this section, the monopole distributions, phase diagrams, transport responses, and device behavior should therefore be understood as theoretical predictions based primarily on Monte Carlo and entropy-model calculations. The aim is to stabilize an excess of a single sign of magnetic charge while retaining monopole mobility. In this restricted but useful analogy, the resulting state plays a role similar to a two-dimensional electron gas (2DEG): the interface supplies a finite carrier density, while the frustrated spin-ice manifold provides the medium through which the carriers move.

### 5.1. Interfacial Generation of a Two-Dimensional Magnetic Monopole Gas

**Figure 5:**
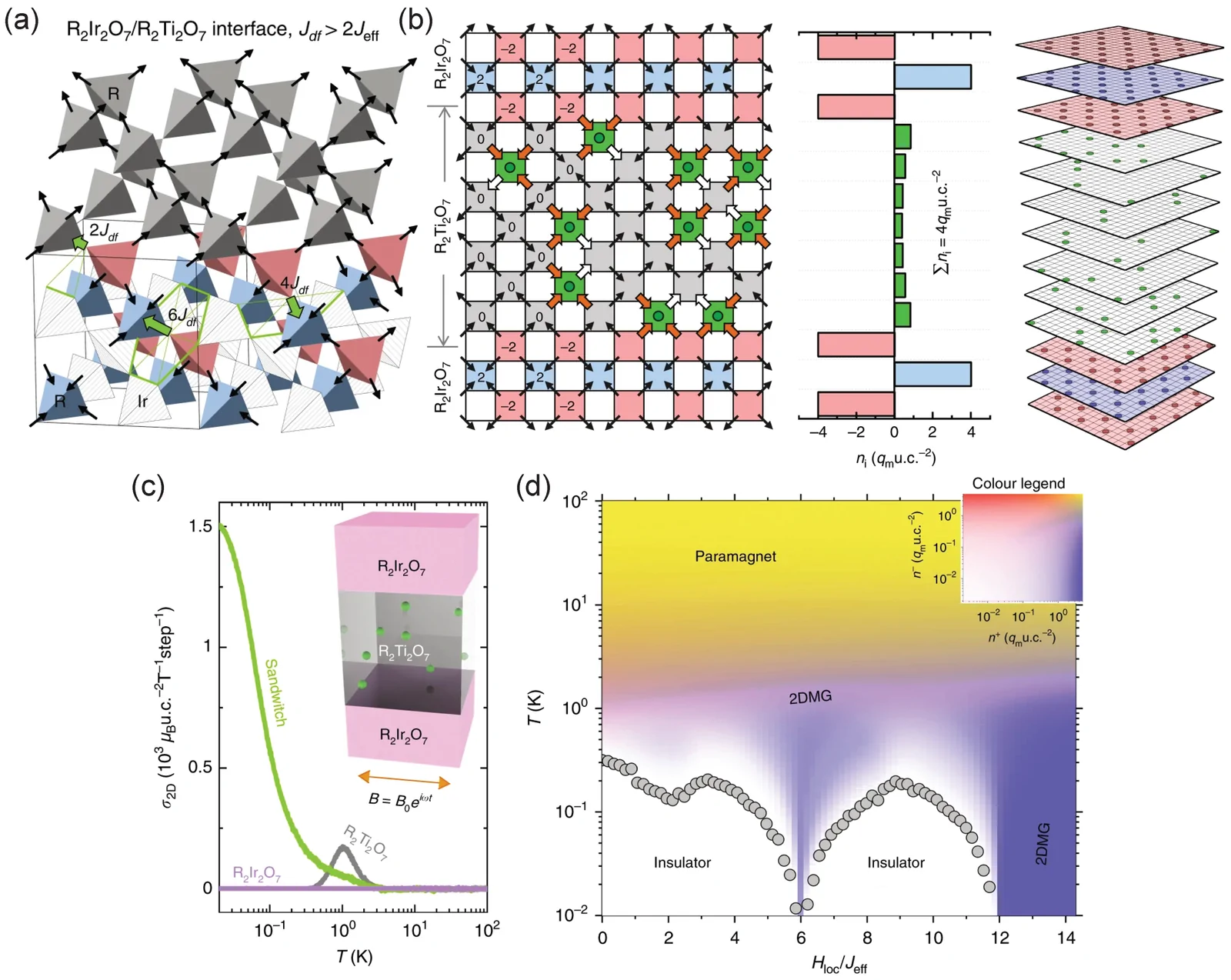
Two-dimensional magnetic monopole gas in pyrochlore oxide heterostructures. (**a**) Schematic of an exchange-coupled $R_{2}$${\mathrm{Ir}}_{2}$$O_{7}$/$R_{2}$${\mathrm{Ti}}_{2}$$O_{7}$ interface, where the Ir-induced boundary condition injects a net magnetic flux into the spin-ice layer. The black arrows denote the local rare earth spins, while the green arrows denote the local d-f interactions. (**b**) Representative Monte Carlo snapshot and layer-resolved monopole profile for an $R_{2}$${\mathrm{Ir}}_{2}$$O_{7}$/$R_{2}$${\mathrm{Ti}}_{2}$$O_{7}$/$R_{2}$${\mathrm{Ir}}_{2}$$O_{7}$ trilayer, showing that same-sign monopoles are concentrated near the interfaces. (**c**) Calculated alternating-current (AC) monopole sheet conductivity, demonstrating metallic-like monopole transport in the heterostructure while the individual constituents become monopole insulating at low temperature. (**d**) Calculated temperature–$H_{\mathrm{loc}}/J_{\mathrm{eff}}$ phase diagram for the (001) heterostructure, showing the parameter regimes in which the 2DMG is stabilized. Adapted from Ref. [80].

The basic design of the 2DMG is illustrated in Figure 5a. The heterostructure consists of a spin-ice $R_{2}$${\mathrm{Ti}}_{2}$$O_{7}$ layer sandwiched between two AIAO pyrochlore iridate $R_{2}$${\mathrm{Ir}}_{2}$$O_{7}$ layers. Because the two materials share the same pyrochlore crystal structure, the corner-sharing tetrahedral network remains continuous across the interface. The Ir magnetic order can therefore impose a well-defined magnetic boundary condition on the rare-earth moments in the adjacent spin-ice layer through the *d*–*f* interaction. For sufficiently strong interfacial coupling, the rare-earth moments within the iridate are locked to the AIAO background, whereas the moments farther inside the titanate retain the frustrated spin-ice interaction.

The essential physics arises from the magnetic flux imposed by the iridate boundaries on the intervening spin ice. For the appropriate AIAO boundary configuration, the ice rule cannot be satisfied on every tetrahedron, as illustrated in Figure 5a; a finite number of monopoles is therefore required within the spin-ice slab. Unlike thermally generated pairs in bulk spin ice, these boundary-induced monopoles all carry the same sign, producing a nonzero net magnetic charge. Reversing the AIAO boundary condition reverses the monopole polarity [80]. The monopoles nevertheless remain mobile: in the nearest-neighbor model, a spin flip can transfer a 3I1O defect to a neighboring 2I2O tetrahedron without changing the total number of monopoles or the energy. The heterostructure therefore combines a finite, charge-polarized monopole population with energetically degenerate hopping, the two ingredients required for monopole-metallic behavior.

### 5.2. Monte Carlo Simulations, Monopole Transport, and Phase Diagram

The proposed state was investigated using single-spin flip Monte Carlo simulations of the heterostructure [80]. As in the preceding section, the ordered Ir sublattice is treated as a static background, while the rare-earth Ising moments are governed by the competition between $J_{\mathrm{eff}}$ and the local field $H_{\mathrm{loc}}=6J_{df}$. The ratio $H_{\mathrm{loc}}/J_{\mathrm{eff}}$ is therefore the principal material parameter. Annealing from high temperature allows the monopole density, spatial distribution, phase stability, and magnetic transport to be obtained without imposing a low-temperature spin configuration.

A representative low-temperature snapshot for a (001)-oriented trilayer with $H_{\mathrm{loc}}/J_{\mathrm{eff}}=14$ is shown in Figure 5b. The spin-ice layer contains only one sign of singly charged monopoles, with an integrated charge fixed by the boundary condition at $4q_{M}$ per unit-cell area and independent of the constituent thicknesses. The layer-resolved profile also reveals a nonuniform distribution: the monopoles are concentrated near the interfaces and decay rapidly toward the interior. This observation establishes the quasi-two-dimensional character of the gas and, as discussed below, poses an important question because nearest-neighbor monopole hopping itself is energetically degenerate.

The thickness independence is important for distinguishing the 2DMG from a finite-size effect. The monopole number sheet density is 4 monopoles per in-plane unit-cell area for the (001) sandwich and does not decrease when the titanate slab is made thicker [80]. Increasing the $R_{2}$${\mathrm{Ti}}_{2}$$O_{7}$ thickness therefore does not drive a 2D-to-3D crossover; it simply adds a bulk-like spin-ice interior beyond the interfacial gas. The monopole profile remains localized within only a few unit cells of the interface, with a characteristic confinement scale of roughly 2–4 unit cells depending on how the profile width is defined, followed by a rapidly decaying tail [80,81]. Thus, even for a macroscopically thick titanate layer, the boundary-induced sheet charge and the interfacial 2DMG remain present rather than expanding into a three-dimensional monopole gas.

A finite monopole density is useful only if the charges remain mobile. To test this, an oscillating longitudinal magnetic field, $B_{0}e^{-i\omega t}$, was applied in the simulation and the time-dependent magnetization was used to obtain the monopole sheet conductivity, $\sigma_{2D}={\left(B_{0}S\right)}^{-1}dM/dt$∼$J_{m}/B_{0}$, where *S* is the interfacial area and $J_{m}$ is the magnetic charge current. As shown in Figure 5c, the bulk titanate and iridate both become monopole insulating at low temperature, whereas the trilayer retains a large AC monopole conductivity that increases upon cooling. The contrast arises because the heterostructure contains a finite ground-state population of mobile charges imposed by its boundaries, so transport does not require thermal creation of additional monopole–antimonopole pairs. The result is a magnetic analogue of a conducting interface between insulating constituents. The analogy is specifically to AC transport: a finite sample cannot sustain a steady equilibrium direct-current (DC) magnetic charge current because continued monopole motion changes the sample magnetization.

The stability of the conducting state depends strongly on $H_{\mathrm{loc}}/J_{\mathrm{eff}}$, as shown in Figure 5d. For the (001) geometry, a ground-state 2DMG appears for $H_{\mathrm{loc}}/J_{\mathrm{eff}}>12$. In the intermediate ranges $6<H_{\mathrm{loc}}/J_{\mathrm{eff}}<12$ and $2<H_{\mathrm{loc}}/J_{\mathrm{eff}}<6$, the low-temperature state is monopole insulating, but a mobile charged 2DMG emerges over a finite-temperature window. The boundary at $H_{\mathrm{loc}}/J_{\mathrm{eff}}=6$ separates the fragmented and AIAO regimes of the rare-earth moments and is accompanied by especially strong competition between configurations. Thus, the 2DMG occupies a broad region of the temperature–interaction phase diagram even when it is not the absolute ground state.

The requirement is substantially relaxed for a (111)-oriented heterostructure. Simulations yield a 2DMG-K1 ground state for $3<H_{\mathrm{loc}}/J_{\mathrm{eff}}<6$ with sheet density $n_{0}^{111}$, and a 2DMG-K2 state for $H_{\mathrm{loc}}/J_{\mathrm{eff}}>6$ with total sheet density $3n_{0}^{111}$, including additional interfacial charges. The experimentally inferred value $H_{\mathrm{loc}}/J_{\mathrm{eff}}\approx 4.5$ for ${\mathrm{Ho}}_{2}$${\mathrm{Ir}}_{2}$$O_{7}$ lies within the 2DMG-K1 regime, indicating that the required coupling is compatible with realistic pyrochlore materials. Similar behavior for kagome- and triangular-terminated (111) interfaces further suggests that the effect is controlled by the boundary condition rather than by a single termination geometry.

The reduced threshold for the (111) orientation can be understood primarily from the orientation-dependent interfacial coordination and the resulting ability of the iridate to maintain the required rare-earth polarization. In bulk $R_{2}$${\mathrm{Ir}}_{2}$$O_{7}$, the local exchange field $H_{\mathrm{loc}}=6J_{df}$ originates from the six nearest-neighbor Ir moments surrounding a rare-earth ion. At a (001) interface, this sixfold coordination is truncated: the two interfacial rare-earth layers experience only $2H_{\mathrm{loc}}/3$ and $H_{\mathrm{loc}}/3$, respectively, of the bulk field [80]. A substantially larger bulk $H_{\mathrm{loc}}$ is therefore needed to impose the polarized boundary condition required for a ground-state 2DMG. The (111) cut is geometrically different because the pyrochlore lattice separates into alternating kagome and triangular spin layers. For the kagome-terminated structure, the boundary condition can be maintained by the polarization of the triangular spin sublattice, corresponding to one of the four spins of each tetrahedron, while retaining a mobile monopole layer in the adjacent spin ice. This difference is especially clear in the fragmented regime: for the (001) sandwich, $2<H_{\mathrm{loc}}/J_{\mathrm{eff}}<6$ produces pinned fragmented charges at the boundary and no mobile ground-state 2DMG, whereas the (111) kagome-terminated structure supports the mobile 2DMG-K1 phase already for $3<H_{\mathrm{loc}}/J_{\mathrm{eff}}<6$ [80]. Thus, the lower (111) threshold is primarily an interfacial-bond and boundary-polarization effect. It should not be attributed mainly to Dirac-string geometry, because the orientation dependence already appears in the nearest-neighbor model, where moving a monopole between neighboring 3I1O and 2I2O tetrahedra is energetically degenerate and the associated Dirac string has no energetic string tension.

### 5.3. Entropy-Induced Two-Dimensional Confinement

The monopole distribution in Figure 5b raises a fundamental question about its dimensionality. The monopoles are strongly concentrated near the $R_{2}$${\mathrm{Ir}}_{2}$$O_{7}$/$R_{2}$${\mathrm{Ti}}_{2}$$O_{7}$ interface, with their density rapidly decreasing toward the interior of the spin-ice layer. However, in the nearest-neighbor spin-ice model, monopole hopping is energetically degenerate: a monopole can move between neighboring tetrahedra through successive spin flips without changing the number of monopoles or the total energy. Why, then, do the monopoles remain near the interface rather than spreading throughout the three-dimensional spin-ice layer?

More generally, theoretical studies of confined spin ice have shown that open boundaries and modified surface couplings can qualitatively reorganize magnetic charge, including two-dimensional surface-charge ordering and boundary-sensitive Coulomb phases [82,83]. The 2DMG confinement problem differs microscopically, but it belongs to the same broader class of boundary-controlled spin-ice phenomena.

**Figure 6:**
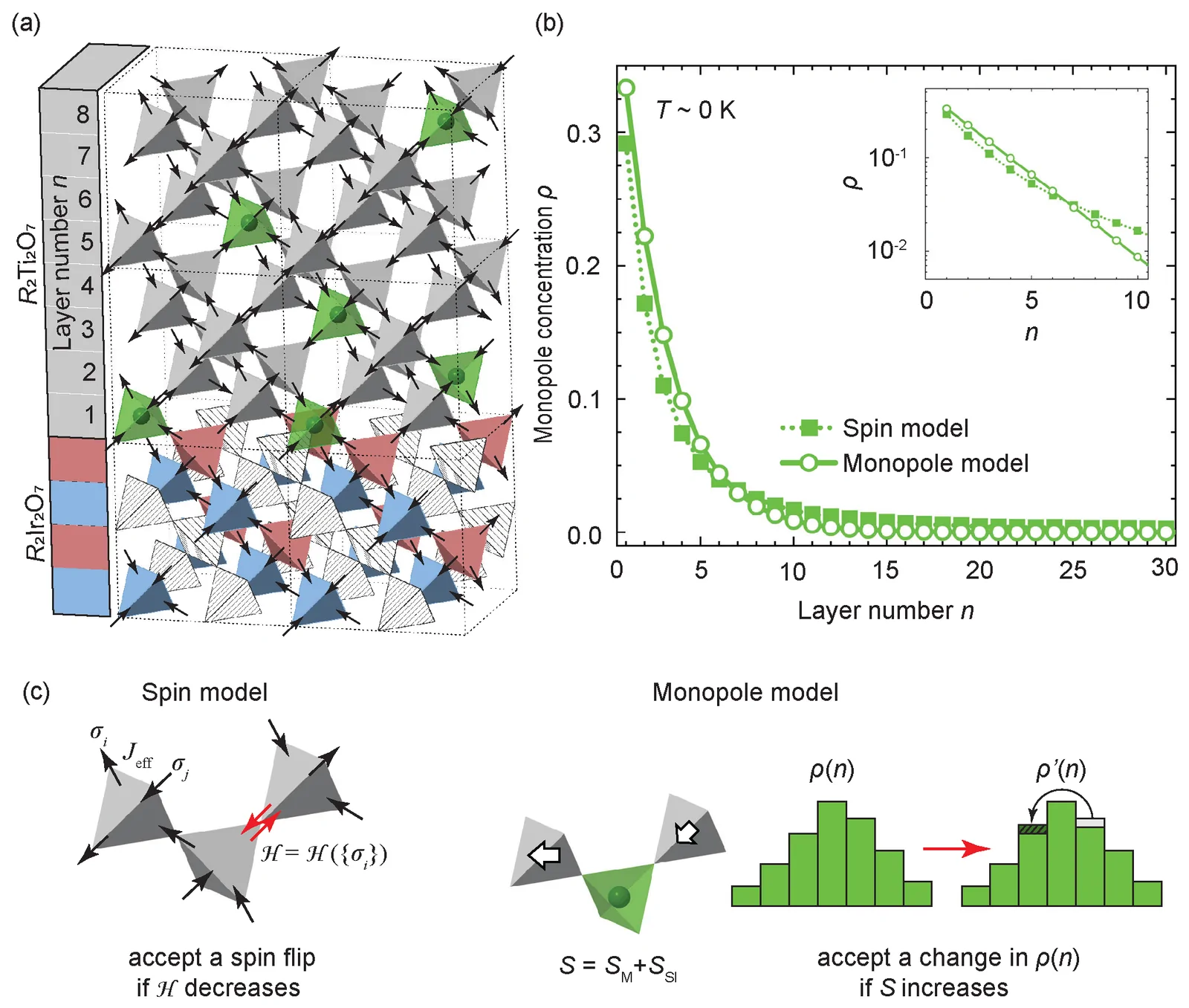
Entropy-induced two-dimensional confinement of the monopole gas. (**a**) Layer-resolved monopole distribution near an $R_{2}$${\mathrm{Ti}}_{2}$$O_{7}$/$R_{2}$${\mathrm{Ir}}_{2}$$O_{7}$ interface, illustrating the enhanced monopole concentration near the boundary. Black arrows denote the local rare earth spins. The green spheres denotes the 3I1O magnetic monopoles. (**b**) Monopole concentration ρ(n) at *T*∼0 obtained independently from the microscopic spin Monte Carlo model and from entropy maximization in the monopole model. The inset shows the approximately exponential decay on a logarithmic scale. (**c**) Schematic comparison of the two calculations: the spin model samples microscopic spin configurations according to their energy, whereas the monopole model varies ρ(n) to maximize the total entropy $S=S_{M}+S_{\mathrm{SI}}$. Adapted from Ref. [81].

The missing ingredient is entropy. Consider moving a monopole several layers away from the interface, as illustrated in Figure 6a. The boundary condition imposed by the ordered iridate fixes the magnetic flux between the interface and the monopole, forcing the intervening spin-ice layers into an essentially polarized configuration and removing their 2I2O degeneracy. If the monopole instead resides close to the interface, the interior layers are released from this constraint and can explore the macroscopically degenerate spin-ice manifold. Although the two configurations can have the same energy, the interface-confined configuration has greater spin-ice entropy and is therefore thermodynamically favored.

The confinement is opposed by the positional entropy of the monopoles themselves: allowing a monopole to explore more sites and layers increases its random walk entropy and favors delocalization. The equilibrium profile therefore reflects a competition between spin-ice configurational entropy, which favors confinement, and monopole random walk entropy, which favors spreading. In the entropy-based model this competition is written as

(9) $$S\left[\rho \left(n\right)\right]=-\sum_{n}k_{B}\rho \left(n\right)\ln\rho \left(n\right)+\sum_{n}2s_{0}\left[1-M\left(n\right)\right],$$

where

(10) $$s_{0}=\frac{k_{B}}{2}\ln\left(\frac{3}{2}\right)$$

is the Pauling residual entropy per spin of an unpolarized spin-ice region, ρ(n) is the monopole concentration in tetrahedral layer *n*, and

(11) $$M\left(n\right)=1-\sum_{m<n}\rho \left(m\right)$$

describes the normalized polarization of layer *n*. Importantly, the model does not assign the constant value $s_{0}$ uniformly throughout the spin-ice volume. Rather, the local spin-ice entropy is weighted by the factor 1−M(n) in Equation (9). For an unpolarized layer, M(n)=0, and the full Pauling entropy $s_{0}$ per spin is retained; for a fully polarized layer, M(n)=1, and the local configurational entropy vanishes. Partially polarized layers interpolate between these two limits. Thus, the reduction in local entropy caused by interfacial polarization is explicitly included in the entropy functional.

Equations (9)–(11) therefore make explicit the competition between monopole positional entropy and the residual entropy of the spin-ice background. The first term, $S_{M}$, is the monopole random walk entropy and favors occupation of many layers. The second, $S_{\mathrm{SI}}$, is the residual configurational entropy of the spin-ice background and favors keeping the monopoles near the interface so that a larger volume remains unpolarized. Their competition determines the equilibrium profile.

Figure 6c compares two independent calculations of this profile. The spin model samples microscopic rare-earth configurations using single-spin flip Monte Carlo dynamics and extracts ρ(n) from approximately 4000 equilibrated snapshots. The monopole model instead discards the microscopic spins and varies ρ(n) directly to maximize Equation (9) at fixed total monopole number. The two methods therefore optimize different variables, microscopic spin energy in one case and emergent monopole entropy in the other.

Despite these different starting points, the two methods produce nearly the same distribution, as shown in Figure 6b. The concentration is about one third in the first tetrahedral layer and falls to roughly 10% of that value within the first eight layers, with an approximately exponential decay visible in the inset. Agreement between the microscopic energy-based calculation and the entropy maximization provides quantitative evidence that the quasi-two-dimensional profile is entropic in origin within the nearest-neighbor model.

Long-range dipolar interactions remove the exact energetic degeneracy of monopole motion and therefore modify this idealized picture. In the dipolar model, monopole–monopole Coulomb interactions can favor additional spatial ordering at the lowest temperatures. Nevertheless, the 2DMG remains robust, and at finite temperature the configurational entropy continues to compete significantly with the dipolar energy. The entropy-induced confinement should therefore be viewed as the dominant mechanism in the nearest-neighbor limit and as an important finite-temperature contribution in more realistic dipolar spin ice [80,81].

The same argument shows that a symmetric trilayer is not required. At a single $R_{2}$${\mathrm{Ir}}_{2}$$O_{7}$/$R_{2}$${\mathrm{Ti}}_{2}$$O_{7}$ interface, creating the charged interfacial state carries an energy cost proportional to the interface area, whereas restoring the spin-ice degeneracy produces an entropy gain proportional to the three-dimensional spin-ice volume. The balance is set by F=U−TS: at strictly T=0 the energy cost dominates, whereas above a critical temperature the entropic gain outweighs it and stabilizes the charged interfacial monopole gas. Because the energy scales with area while the entropy scales with area times thickness, the critical temperature decreases approximately as the inverse spin-ice thickness. For a thickness of about 1 mm, the estimated scale is only ∼700 nK. A single interface can therefore host the entropy-stabilized 2DMG over essentially the entire experimentally accessible temperature range [80,81].

## 6. Toward Monopole-Based Devices

**Figure 7:**
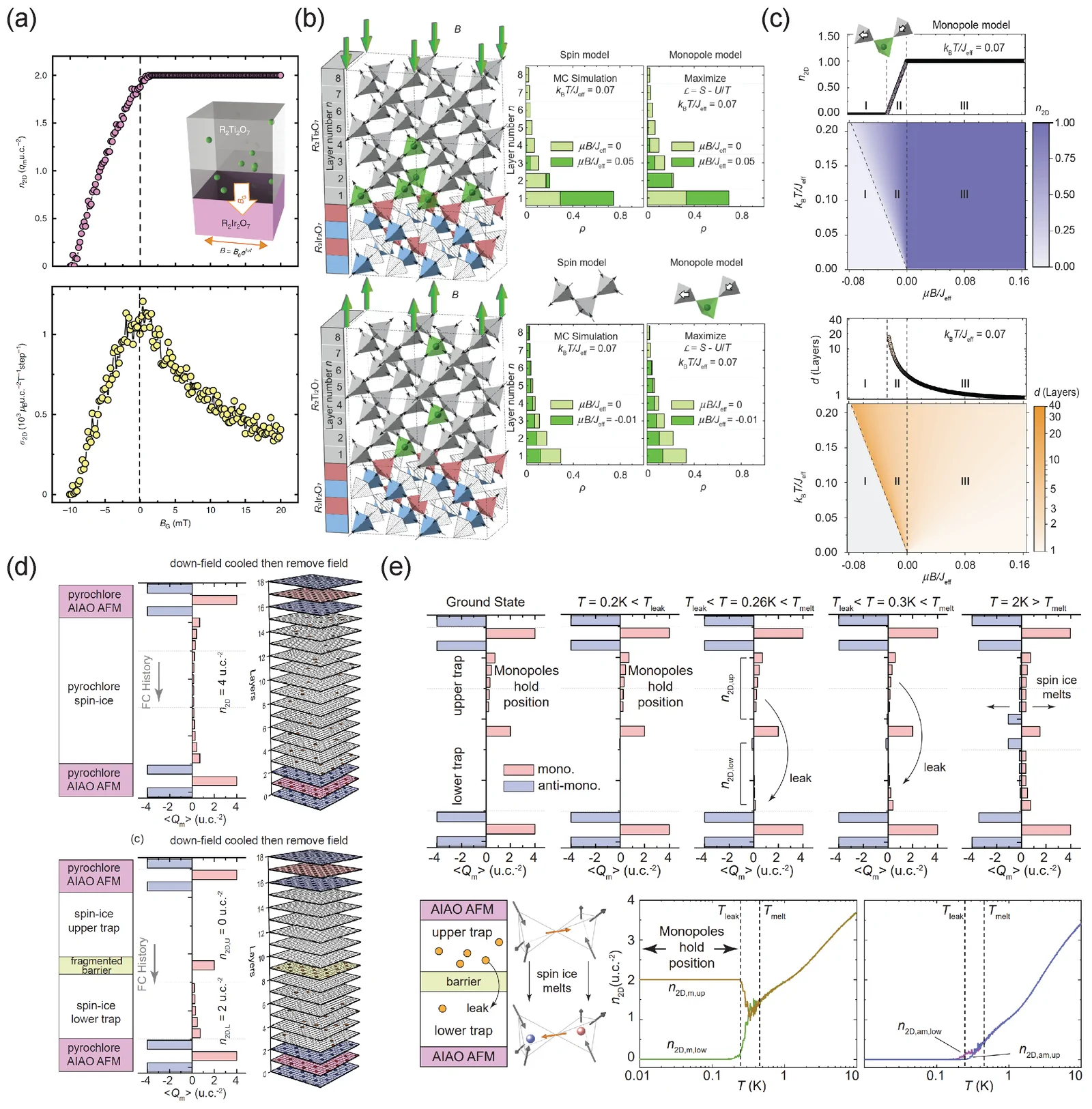
Field control and device concepts based on two-dimensional magnetic monopole gases. (**a**) Proposed magnetic field-controlled monopole transistor and calculated magnetic field dependence of the monopole sheet density and AC sheet conductivity at a single $R_{2}$${\mathrm{Ir}}_{2}$$O_{7}$/$R_{2}$${\mathrm{Ti}}_{2}$$O_{7}$ interface. (**b**) Calculated monopole sheet density and confinement depth as functions of perpendicular magnetic field, comparing the spin Monte Carlo and entropy-based monopole models. The black arrows denote the local rare earth spin, the green arrows denote the applied magnetic field. The green spheres denote the magnetic monopoles. (**c**) Corresponding temperature–field maps of the monopole sheet density and confinement depth, showing the convergence of the field-response regimes toward T=0 and B=0. Phase I is the regime where monopoles are fully depleted; Phase II is the regime where monopoles are partially depleted; Phase III is the regime where the monopoles are pushed toward the interface. (**d**) Monopole-trap heterostructure containing a fragmented pyrochlore iridate barrier. Opposite field-cooling histories localize the monopoles in the lower or upper trap, producing bistable position-encoded states after field removal. (**e**) Zero-field warm-up of a trapped state, showing monopole retention below $T_{\mathrm{leak}}$, redistribution between the traps above $T_{\mathrm{leak}}$, and thermally generated monopole–antimonopole pairs above $T_{\mathrm{melt}}$. Adapted from Refs. [80,81,84].

Once a magnetically charged 2DMG can be stabilized and confined, the problem naturally shifts from observing emergent monopoles to controlling them as functional degrees of freedom. Because a perpendicular magnetic field couples directly to the monopole charge, it can tune the interfacial carrier density, confinement depth, and magnetic conductivity. Heterostructure design can further reshape the monopole free energy landscape and stabilize distinct spatial configurations. Figure 7 summarizes two proposed device concepts based on these capabilities: a magnetic field-effect transistor (MFET) for monopoles, in which a magnetic field controls a conducting 2DMG channel, and a monopole trap, in which information is encoded in bistable monopole positions. Both concepts are presently theoretical and should be viewed as physically motivated targets for future materials and device experiments rather than demonstrated technologies.

### 6.1. Magnetic Field-Effect Transistor for Monopoles

The analogy between the 2DMG and the 2DEG naturally motivates the MFET illustrated in Figure 7a [80]. Unlike a conventional electronic field-effect transistor, no electric field or electrostatic gate is used in the MFET. A single $R_{2}$${\mathrm{Ir}}_{2}$$O_{7}$/$R_{2}$${\mathrm{Ti}}_{2}$$O_{7}$ interface hosts the monopole channel, while a perpendicular magnetic field $B_{G}$ acts as the gate variable and couples directly to the emergent magnetic charge. One field polarity attracts monopoles toward the interface; the opposite polarity drives them away. The magnetic gate therefore controls the monopole sheet density, confinement depth, and AC conductivity.

The calculated response in Figure 7a shows both depletion and accumulation. A negative gate field progressively removes monopoles from the interface, reducing the sheet density and ultimately suppressing the AC monopole conductivity. A positive field instead confines the monopoles more strongly at the interface. The conductivity is nonmonotonic, however: sufficiently strong positive fields compress the monopoles into the first interfacial layer and reduce the number of available hopping sites. Maximum conductivity therefore occurs at an intermediate distribution rather than at the largest possible interfacial density.

The thermodynamic origin of this field control can be understood from the entropy description developed in the previous section [81]. At finite temperature and perpendicular magnetic field, the equilibrium monopole distribution ρ(n) minimizes the free energy, or equivalently maximizes the modified entropy functional

(12) $$L\left[\rho \left(n\right);B,T\right]=S\left[\rho \left(n\right)\right]-\sum_{n}\frac{k_{B}q_{M}\rho \left(n\right)naB}{T},$$

where S[ρ(n)] is the zero-field entropy functional introduced above, $q_{M}$ is the monopole charge, *a* is the pyrochlore lattice constant, and *n* labels tetrahedral layers from the interface. The field-dependent term in Equation (12) biases monopoles according to their distance from the interface, while the entropy favors the distribution discussed in Figure 6. Field control of the 2DMG is therefore a direct competition between magnetic energy and the entropic confinement mechanism.

The resulting field dependence is summarized in Figure 7b. At T=0.1 K, three regimes appear, where μ denotes the rare-earth magnetic moment. For $\mu B/J_{\mathrm{eff}}<-0.027$ (region I), the interfacial monopole population is depleted. For $-0.027<\mu B/J_{\mathrm{eff}}<0$ (region II), a lower-density and more weakly confined gas remains. For $\mu B/J_{\mathrm{eff}}>0$ (region III), the full sheet density is retained, and the monopoles are pushed progressively closer to the interface. The close agreement between the spin Monte Carlo and entropy-based calculations shows that the same entropy model that explains confinement also captures the field response.

Figure 7c extends this result over temperature and field. The same three regimes are visible in both the monopole sheet density and the confinement depth, and their boundaries converge toward T=0 and B=0. The enhanced low-temperature field sensitivity follows from the B/T scaling in the modified entropy functional: as *T* decreases, a progressively smaller field can reorganize an otherwise energetically degenerate monopole distribution.

Together, Figure 7a–c establish the central MFET principle: the interface supplies a monopole-conducting channel, while a perpendicular magnetic field controls its carrier density, confinement, and AC conductivity. The functionality is therefore transistor-like, but the active carriers are emergent magnetic charges and the gate response is governed by the competition between magnetic energy and configurational entropy.

Importantly, the single-interface MFET is not predicted to show intrinsic nonvolatile hysteresis in this equilibrium model [80,81]. At a given *B* and *T*, the monopole distribution relaxes toward the corresponding equilibrium state, and after the magnetic gate is removed it returns to the zero-field distribution. A single MFET is therefore a field-tunable monopole conductor rather than, by itself, a magnetic memory element. Hysteresis requires an additional metastable barrier, which motivates the monopole-trap architecture discussed next. There, the fragmented iridate barrier inhibits monopole transfer between two traps and produces bistable, hysteretic switching that can retain a position-encoded state after the writing field is removed [84].

### 6.2. Monopole Traps for Position-Based Information Encoding

A second device concept uses monopole *position*, rather than monopole density, as the information variable [84]. The architecture is illustrated in Figure 7d. Two spin-ice regions are separated by a fragmented pyrochlore iridate layer, while AIAO pyrochlore iridate layers terminate the heterostructure on both sides. The two spin-ice regions therefore form an upper and a lower monopole trap, and the central fragmented layer acts as a barrier to monopole propagation.

The fragmented phase is particularly suitable as a monopole barrier. A 2I2O region cannot effectively trap monopoles because monopole hopping through the spin-ice manifold is energetically degenerate. A rigid AIAO region, on the other hand, cannot readily accommodate the Dirac string associated with monopole motion. The fragmented state provides the required intermediate behavior: moving a monopole into the barrier produces an energetically unfavorable defect, while its remaining configurational freedom permits the spin rearrangements associated with the Dirac string. The barrier therefore suppresses spontaneous monopole transfer while preserving the microscopic degrees of freedom required for monopole motion [84].

As shown in Figure 7d, the resulting structure possesses two stable monopole configurations. Cooling under one field direction places the monopoles predominantly in the lower trap, whereas reversing the cooling field places them in the upper trap. After the magnetic field is removed, the fragmented barrier prevents the monopoles from returning to the symmetric equilibrium distribution. The field-cooling history is therefore retained as the spatial position of the monopoles, providing a bistable, nonvolatile information state. The trap containing monopoles develops an extended spin texture, whereas the monopole-free trap remains nearly polarized by the neighboring AIAO boundary conditions. Consequently, switching the monopoles between the two traps also reverses the net magnetization of the heterostructure, providing a possible nondestructive magnetic readout of the encoded state [84].

The thermal stability of the encoded state is summarized in Figure 7e. Below $T_{\mathrm{leak}}$∼0.22 K, the monopoles remain in the trap in which they were written. Above $T_{\mathrm{leak}}$, they begin to cross the fragmented barrier and redistribute between the two traps, progressively erasing the positional information. A second scale, $T_{\mathrm{melt}}$∼0.45 K, marks the onset of thermally generated monopole–antimonopole pairs throughout the spin-ice regions. The device therefore exhibits two distinct thermal processes: leakage of the encoded monopoles and, at higher temperature, breakdown of the underlying ice-rule manifold [84].

This loss of memory is again controlled by energy–entropy competition. The fragmented barrier stabilizes a one-trap configuration energetically, whereas distributing monopoles between both traps increases configurational entropy. Rising temperature therefore favors redistribution and eventually overcomes the trapping barrier. The role of entropy is consequently different from the interfacial confinement problem: spin-ice entropy confines the 2DMG near an interface, while the additional positional entropy of the two-trap system favors leakage between the traps.

The monopole positions can also be switched isothermally by sweeping the external magnetic field. Below $T_{\mathrm{leak}}$, the upper- and lower-trap populations exhibit strongly anticorrelated hysteretic switching, demonstrating reversible transfer of monopoles through the fragmented barrier. As the temperature approaches $T_{\mathrm{leak}}$, the hysteresis narrows because thermal fluctuations assist barrier crossing; near $T_{\mathrm{melt}}$ the switching becomes nearly reversible, and above the melting temperature the bistability disappears. Repeated field cycles produce reproducible switching, while the associated bistable magnetization provides a potential readout signal using spatially resolved magnetic probes such as scanning SQUID microscopy [84].

Figure 7 thus illustrates two complementary routes to monopole functionality. Panels Figure 7a–c use monopole density and transport as the controllable variables, whereas Figure 7d,e use monopole position as a memory variable. Both concepts rely on the same underlying ingredients developed throughout this review: geometric frustration, emergent magnetic charge, interface-imposed boundary conditions, and entropy.

## 7. Outlook

The developments reviewed above suggest a broader future program than the realization of the 2DMG alone. Classical spin ice still presents unresolved questions concerning its true low-temperature equilibrium, monopole kinetics, and the role of disorder. Pyrochlore iridates offer a complementary opportunity to test how competing interactions redistribute entropy and stabilize mixed magnetic states. Interface engineering then adds a new level of control by allowing magnetic charge and dimensionality to be designed. Beyond the classical regime, quantum spin ice and artificial spin ice provide distinct routes for extending these ideas to coherent quasiparticles and lithographically programmable frustrated systems. The most important future directions can therefore be organized from bulk frustrated magnets to engineered monopole matter.

### 7.1. Classical Spin Ice: Equilibrium, Dynamics, and Disorder

Despite the maturity of classical spin-ice physics, the lowest-temperature regime of canonical compounds such as ${\mathrm{Dy}}_{2}$${\mathrm{Ti}}_{2}$$O_{7}$ and ${\mathrm{Ho}}_{2}$${\mathrm{Ti}}_{2}$$O_{7}$ remains an important frontier. The ideal dipolar spin-ice model predicts that interactions beyond the nearest-neighbor ice-rule manifold can eventually select ordered states, yet experimentally the spins become extremely slow before equilibrium is easily reached [35,36,37]. Distinguishing an intrinsic thermodynamic transition from kinetic arrest, weak disorder, hyperfine effects, or small corrections to the effective Hamiltonian remains essential for connecting the Pauling entropy regime to the true equilibrium ground state. Long-time calorimetry, neutron scattering, magnetostriction, and local magnetic probes performed under carefully controlled thermal protocols may help resolve this problem.

A related challenge is to understand monopole dynamics quantitatively across many time scales. Monopole motion is constrained not only by Coulomb interactions and Dirac strings [30] but also by impurities [85] and by surfaces or strain that modify the spin-ice manifold [82]. These effects can act as traps or bottlenecks and may explain part of the broad distribution of relaxation times observed at low temperature. A predictive theory connecting microscopic defects to macroscopic magnetic relaxation would be valuable both for fundamental spin-ice thermodynamics and for any future attempt to use monopole transport functionally.

### 7.2. Pyrochlore Iridates and Entropic Phase Control

Pyrochlore iridates offer a particularly direct setting for testing entropy-driven phase selection because the ratio $H_{\mathrm{loc}}/J_{\mathrm{eff}}$ links rare-earth frustration to an ordered Ir background. An immediate experimental goal is to establish whether the predicted ${\mathrm{EE}}_{I-\mathrm{II}}$ and ${\mathrm{EE}}_{\mathrm{II}-\mathrm{III}}$ regimes can be resolved through correlated anomalies in specific heat, entropy, susceptibility, and neutron scattering [25]. Chemical substitution, hydrostatic pressure, epitaxial strain, and controlled disorder could provide complementary means of tuning the effective *d*–*f* coupling through the relevant phase boundaries.

A second open question concerns the feedback between the two magnetic sublattices. Treating the Ir moments as a static background is well justified on the rare-earth temperature scale in the simplified model, but real materials may exhibit domain effects, weak Ir dynamics, strain dependence, and coupling between magnetic and electronic degrees of freedom. Thin films make magnetic-domain populations, epitaxial strain, and growth-induced structural variations especially important [86,87,88]. Determining when the static-background approximation remains valid, and when coupled rare-earth–Ir dynamics must be retained explicitly, is therefore important for both the EE-phase problem and the 2DMG proposal.

### 7.3. Experimental Realization and Detection of Two-Dimensional Monopole Gases

Direct experimental realization of a 2DMG remains the central materials challenge for the interface-engineered part of this review. Epitaxial spin-ice films have already demonstrated that confinement and strain can strongly modify spin-ice thermodynamics, including the removal of residual entropy and phase transitions in the few-monolayer limit [89,90]. Epitaxial pyrochlore films have been realized by off-axis sputtering and pulsed laser deposition (PLD), while thermodynamic studies have clarified the narrow growth window and limitations of conventional physical vapor deposition routes [88,91,92]. The proposed 2DMG nevertheless places unusually stringent requirements on crystalline continuity, cation order, oxygen stoichiometry, interface roughness, and magnetic-domain control. The relevant *d*–*f* exchange must be strong and spatially well defined while the titanate layer retains the local Ising anisotropy and spin-ice correlations required for mobile monopoles. Orientation is also a practical design variable because the calculations predict substantially different thresholds for (001) and (111) interfaces.

Recent pyrochlore heterostructure experiments provide important precedents for this program, although they do not yet realize the proposed 2DMG. In ${\mathrm{Bi}}_{2}$${\mathrm{Ir}}_{2}$$O_{7}$/${\mathrm{Dy}}_{2}$${\mathrm{Ti}}_{2}$$O_{7}$, ice-rule breaking in the spin-ice layer produces an anomalous magnetoresistance in the adjacent conductor [93]; a ${\mathrm{Bi}}_{2}$${\mathrm{Rh}}_{2}$$O_{7}$/${\mathrm{Dy}}_{2}$${\mathrm{Ti}}_{2}$$O_{7}$ interface similarly converts field-driven spin-ice transitions into longitudinal and Hall transport signatures [94]. Very recent work has also demonstrated atomically sharp ${\mathrm{Dy}}_{2}$${\mathrm{Ti}}_{2}$$O_{7}$/${\mathrm{Eu}}_{2}$${\mathrm{Ir}}_{2}$$O_{7}$ pyrochlore interfaces and interfacial electronic anisotropy arising from coupling between spin ice and a Weyl-semimetal state [95,96]. Together, these experiments show that coherent pyrochlore interfaces can transmit information about frustrated spin configurations into measurable electronic responses, strengthening the materials basis for future tests of interface-engineered monopole states.

Detection is equally important because the magnetic charge is confined within only a few layers of an interface. No single existing probe is likely to establish all properties of a 2DMG. Polarized neutron reflectometry can constrain depth-dependent magnetic structures [97], while resonant X-ray scattering can provide element- and order-sensitive information on interfacial magnetism [98]. Scanning SQUID microscopy [99], nitrogen-vacancy (NV) magnetometry [100], and local Hall probes [101] can search for interfacial stray fields and magnetic noise. A compelling demonstration would ideally combine evidence for a nonzero net magnetic charge, the predicted confinement profile, and a dynamic response attributable to monopole motion. Measuring the magnetic field dependence predicted for the MFET geometry would provide an additional stringent test because density, confinement depth, and AC conductivity should change in a correlated manner.

Point defects are expected to perturb monopole motion locally, but the boundary-imposed sheet charge is much more difficult to eliminate. Revell et al. showed that a very dilute level of stuffed spins, about 0.30%, can trap monopoles and strongly affect low-temperature relaxation in ${\mathrm{Dy}}_{2}$${\mathrm{Ti}}_{2}$$O_{7}$ [85]. This concentration is nevertheless small compared with the monopole density imposed by the 2DMG boundary condition. A conventional pyrochlore unit cell contains 16 Ti sites, so 0.30% stuffing corresponds to only ∼0.048 point defects per unit-cell volume. Projected through a 3–4 unit-cell-thick interfacial region, this gives only ∼0.14–0.19 defects per in-plane unit-cell area. Even taking two trapped monopoles per point defect as a deliberately conservative local estimate, the corresponding trapping capacity is only ∼0.3–0.4 monopoles per unit-cell area, compared with the boundary-imposed 2DMG density of 4 monopoles per unit-cell area. Dilute point defects can therefore pin a fraction of the monopoles and reduce mobility, but they cannot absorb the macroscopic boundary-imposed charge. The existence of the charged 2DMG should consequently be substantially more robust to dilute point disorder than its transport properties.

The influence of long-range dipolar interactions, realistic disorder, and finite interfaces should also be explored beyond the nearest-neighbor calculations. The entropy-induced confinement mechanism is particularly transparent in the energetically degenerate limit, whereas real dipolar spin ice introduces energetic correlations among monopoles. Establishing which signatures of the 2DMG survive quantitatively in realistic material-specific Hamiltonians will be crucial before comparing theory directly with experiment [80,81].

### 7.4. Quantum Spin Ice and Quantum Two-Dimensional Monopole Matter

The bulk QSI framework and recent experimental evidence were summarized in Section 2.4. From the perspective of entropy engineering, a key open question is how the quantum-coherent ice manifold responds to surfaces, interfaces, and reduced dimensionality. Unlike classical spin ice, where monopole motion can often be understood as thermally activated hopping of defects through a constrained background, QSI involves coherent tunneling and a dynamical emergent gauge field [38,52]. Interfaces could therefore modify not only quasiparticle confinement but also the emergent photon and spinon spectra.

A particularly interesting possibility is a quantum analogue of the 2DMG. In such a state, the quasiparticles would no longer be classical hopping defects in a static spin background. Quantum tunneling, gauge field dynamics, and coherent quasiparticle propagation could modify both the meaning of monopole confinement and the entropy arguments developed for the classical heterostructure. Interfaces might also act as boundary conditions for emergent electrodynamics, producing confined photon modes or reconstructed fractionalized spectra. Developing microscopic models of quantum spin-ice heterostructures, followed by experimentally realistic predictions for neutron and local noise probes, would be a natural next step. Recent theory of stray-field noise from the emergent photon illustrates one possible local probe [102].

### 7.5. Artificial Spin Ice and Artificial Monopole Matter

The construction, direct imaging, and entropy physics of ASI were summarized in Section 2.5. For the present perspective, its main advantage is that geometry, interaction strengths, disorder, and driving protocols can be designed directly rather than inherited from a bulk crystal [8,55]. This makes ASI a natural test bed for controlled monopole nucleation, propagation, trapping, clocked dynamics, and collective memory at experimentally accessible length and time scales [57,62,65,69].

These capabilities suggest an artificial counterpart to the 2DMG program. Rather than relying on atomic-scale *d*–*f* exchange, an ASI could use patterned boundaries, spatially varying island moments, local field sources, or multilayer geometry to inject a net vertex charge and confine it to a selected region. Such systems would not be microscopic realizations of the same pyrochlore Hamiltonian, but they could test the more general principles of charge injection, entropic versus energetic confinement, monopole-current control, and position-based information encoding. A particularly useful goal would be to design an ASI in which the relative contributions of energetic and entropic confinement can be varied independently and visualized in real space [57,59,70,71,72].

### 7.6. Entropy Engineering and Perspective

A useful way to make entropy engineering experimentally concrete is to separate two control functions: setting the monopole chemical potential, which determines where magnetic charge is energetically favored, and shaping the configurational entropy landscape, which determines how many spin configurations are available to a monopole as it moves. Interfaces naturally provide the first function. In the proposed spin-ice/pyrochlore iridate heterostructure, the interfacial *d*–*f* exchange imposes a magnetic boundary condition with nonzero flux and thereby injects a net monopole charge, while the competition between spin-ice entropy and monopole random walk entropy confines the mobile charge within a few layers of the interface [80,81]. This suggests a direct experimental strategy: fabricate a thickness wedge or a superlattice series with nominally identical interfaces and measure the depth profile, magnetic noise, or transport response as a function of spin-ice thickness, temperature, and field. Because the interfacial boundary condition controls the injected sheet charge whereas the spatial distribution reflects the available configuration space, such a series could separate monopole injection from monopole confinement. Film orientation provides an additional discrete control parameter because different surface terminations and boundary connectivities can produce qualitatively different surface-charge sectors [82,83].

Strain and disorder provide complementary microscopic controls, but they should not be treated as equivalent. Epitaxial strain changes the exchange hierarchy and can lift the degeneracy of the spin-ice manifold: ${\mathrm{Dy}}_{2}$${\mathrm{Ti}}_{2}$$O_{7}$ thin films show a loss of Pauling entropy consistent with strain-induced ordering, and theory likewise finds that strain can smoothly remove the spin-ice entropy [82,89]. A substrate series, or ultimately a piezoelectrically tunable structure, could therefore be used to vary the splitting among nominally degenerate ice configurations and tune the monopole creation energy and effective string tension without changing chemical composition. Dimensional confinement offers a second independent axis: few-monolayer films already exhibit entropy release and phase behavior distinct from bulk spin ice [90]. A combined thickness–strain phase diagram would be particularly informative because it could distinguish entropy changes caused by reduced configuration space from those caused by explicit symmetry breaking. Disorder plays a different role. Even a small concentration of stuffed spins can trap monopoles and strongly slow their motion [85]. Controlled dilute disorder could thus be used as a designed pinning landscape: comparing clean and intentionally disordered samples at the same interface charge density would separate true entropic confinement from kinetic trapping and establish how robust monopole transport is to realistic defects.

Artificial spin ice offers the most direct platform for testing these ideas because the entropy landscape and monopole energetics can be designed separately and then imaged in real space. A vertical height offset can restore the near-degeneracy of square ice and strongly reduce the energetic cost of separating monopoles [59]; engineered defects can act as reproducible monopole injection sites [62]; and three-dimensional architectures can suppress Dirac-string tension and permit steered magnetic charge motion [71]. Combining these ingredients suggests a particularly clean experiment: a thermally active ASI with a fixed monopole injector but a spatially graded height offset or interaction strength. The injector would set the source of magnetic charge, while the gradient would tune the local degeneracy and string tension. Real-space imaging would then give the monopole probability distribution P(r), from which an effective free energy landscape can be inferred through $F_{\mathrm{eff}}\left(r\right)=-k_{B}T\ln P\left(r\right)$. Repeating the measurement while changing only the geometric degeneracy would directly reveal how much of the confinement arises from entropy rather than from an energetic barrier.

These examples suggest that the most useful goal is not simply to increase or decrease entropy globally, but to engineer a spatially varying free energy landscape for magnetic charge. Interfaces can inject charge, thickness and strain can reshape the number and energy of accessible spin configurations, disorder can introduce controlled traps, and artificial architectures can tune degeneracy and string tension almost independently. In this sense, entropy engineering could become an analogue of band engineering for emergent monopoles: the experimentally relevant object is the combined energetic and entropic potential that determines where monopoles are created, where they accumulate, and how easily they move.

## 8. Conclusions

This review has examined spin ice from the perspective of entropy and emergent magnetic charge. In classical spin ice, geometric frustration produces the extensively degenerate 2I2O manifold, the Pauling residual entropy, and thermally activated monopole excitations. Pyrochlore iridates show how this degeneracy can be progressively reorganized by an additional magnetic sublattice, producing 2I2O, fragmented 3I1O/1I3O, and AIAO states with distinct entropic character, as well as finite-temperature entropy-enhanced phases. Quantum spin ice and artificial spin ice extend the same constrained-manifold physics in complementary directions: quantum tunneling converts the classical ice manifold into a coherent quantum state with fractionalized excitations and emergent gauge dynamics, whereas artificial spin ice makes frustration, configurational entropy, and monopole-like defects directly designable and observable.

A central message emerging from these systems is that entropy can play a role beyond that of a thermodynamic diagnostic. In interface-engineered spin-ice heterostructures, boundary conditions can generate a finite magnetic charge density, while the configurational entropy of the spin-ice background favors confinement of the monopoles near the interface. This provides a concrete example in which entropy participates directly in determining the spatial organization and possible functionality of an emergent quasiparticle system. Across classical spin ice, pyrochlore iridates, quantum spin ice, and artificial spin ice, the broader progression is therefore from residual entropy to entropy-driven phase selection, quasiparticle confinement, and ultimately entropy engineering.

Several experimental challenges now stand out. These include resolving the true equilibrium low-temperature state of classical spin ice; testing the predicted entropy-enhanced regimes of pyrochlore iridates through thermodynamic and scattering measurements; obtaining more direct signatures of fractionalized matter and emergent photons in quantum spin ice; and exploiting artificial spin ice for real-space control of entropy and magnetic charge. For interface-engineered monopole matter, the decisive challenge is to combine sufficiently coherent pyrochlore heterostructures with measurements capable of establishing net magnetic charge, interfacial confinement, and monopole dynamics in the same system. Meeting these challenges would determine whether entropy can become a reproducible control parameter for emergent magnetic states rather than simply a consequence of frustration.

## Figures and Tables

**Table 1:** Comparison of magnetic states, entropy, monopole and fractionalized excitations, and experimental signatures across spin-ice platforms.

System	Magnetic States	Entropy	Monopole/ Fractionalized Excitations	Representative Signatures
**Classical spin ice**	2I2O Coulomb phase; field-selected or low-*T* ordered states.	Pauling residual entropy from the degenerate 2I2O manifold.	Thermally activated 3I1O/1I3O charges connected by Dirac strings.	Calorimetry; pinch points; magnetic Wien effect; monopole noise [20,24,28,29].
**Pyrochlore iridates**	2I2O; fragmented (3I1O/1I3O); AIAO; predicted entropy-enhanced phases.	Partial lifting of spin-ice entropy; fragmented dimer entropy; enhanced mixing entropy near phase boundaries.	3I1O/1I3O charges forming a fragmented monopole crystal; mobile charge defects.	Bragg plus diffuse neutron scattering; magnetocaloric entropy change; residual entropy; heat capacity [21,25,26,74].
**Quantum spin ice**	U(1) quantum Coulomb/ spin-liquid regimes.	Quantum mixing removes extensive Pauling entropy; low-*T* entropy reflects emergent gauge modes.	Fractionalized gauge charges (spinons), dual topological monopoles, and an emergent photon.	Neutron continua; modified pinch points; photon-like low-energy modes; thermodynamics [44,45,51,52].
**Artificial spin ice**	Square/kagome ice; engineered Coulomb phases; charge-ordered states.	Geometry-tunable configurational entropy obtained from imaged microstates.	Charged vertex defects linked by reversed-island strings; directly imaged and manipulated.	MFM/XMCD-PEEM/LTEM; vertex statistics; pinch points; real-time monopole motion [55,57,58,59,62].

## Data Availability

No new data were created or analyzed in this study. Data sharing is not applicable.
